# The association of alcohol dependence and consumption during adolescence with depression in young adulthood, in England: a prospective cohort study

**DOI:** 10.1016/S2215-0366(23)00138-4

**Published:** 2023-07

**Authors:** Gemma Hammerton, Gemma Lewis, Jon Heron, Gwen Fernandes, Matthew Hickman, Glyn Lewis

**Affiliations:** a Population Health Sciences, Bristol Medical School, University of Bristol, Bristol, UK; b Medical Research Council Integrative Epidemiology Unit, University of Bristol, Bristol, UK; c Division of Psychiatry, Faculty of Brain Sciences, University College London, London, UK; d Critical Thinking Unit, Public Health Directorate, NHS England, UK; e Injury, Recovery and Inflammation Sciences Unit, School of Medicine, University of Nottingham, Nottingham, UK

## Abstract

**Background:**

The role of alcohol use in the development of depression is unclear. We aimed to investigate whether alcohol dependence, but not high frequency or quantity of consumption, during adolescence increased the risk of depression in young adulthood.

**Methods:**

In this prospective cohort study, we included adolescents who were born to women recruited to the Avon Longitudinal Study of Parents and Children in Avon, UK, with delivery dates between April 1, 1991, and Dec 31, 1992. Alcohol dependence and consumption were measured at about age 16 years, 18 years, 19 years, 21 years, and 23 years using the self-reported Alcohol Use Disorders Identification Test, and at about age 18 years, 21 years, and 23 years using items corresponding to DSM-IV symptoms. The primary outcome was depression at age 24 years, assessed using the Clinical Interview Schedule Revised. Analyses were probit regressions between growth factors for alcohol dependence and consumption and depression, before and after adjustments for confounders: sex, housing tenure, maternal education, maternal depressive symptoms, parents' alcohol use, conduct problems at age 4 years, being bullied from age 12–16 years, and frequency of smoking cigarettes or cannabis. Adolescents were included in analyses if they had data from at least one timepoint for alcohol use and confounders.

**Findings:**

We included 3902 adolescents (2264 [58·0%] female; 1638 [42·0%] male) in our analysis, and 3727 (96·7%) of 3853 participants with data on ethnicity were White. After adjustments, we found a positive association between alcohol dependence at 18 years of age (latent intercept) and depression at 24 years of age (probit coefficient 0·13 [95% CI 0·02 to 0·25]; p=0·019), but no association between rate of change (linear slope) and depression (0·10 [–0·82 to 1·01]; p=0·84). There was no evidence of an association between alcohol consumption and depression (latent intercept probit coefficient –0·01 [–0·06 to 0·03]; p=0·60; linear slope 0·01 [–0·40 to 0·42]; p=0·96) after adjustments.

**Interpretation:**

Psychosocial or behavioural interventions that reduce the risk of alcohol dependence during adolescence could contribute to preventing depression in young adulthood.

**Funding:**

UK Medical Research Council and Alcohol Research UK (grant number MR/L022206/1).

## Introduction

Depression is among the top five contributors to the global burden of disease.^1^ Incidence increases sharply around age 13 years in girls and 16 years in boys and continues to rise into young adulthood, with steeper increases among girls.^2^ There is evidence that rates of adolescent depression are rising in several high-income countries.^3^ Improving the understanding of modifiable risk factors is a priority to inform primary prevention.

Alcohol consumption has fallen among adolescents in most high-income countries in the past 20 years, but this has not led to a reduction in alcohol-related harms among young adults.^4^ Variation is seen across countries,^5^ but in the UK, alcohol use increases sharply between age 15 and 18 years (with limited evidence of gender differences),^6^ stabilises, and then decreases slightly by age 22 years.^7^ Alcohol use and depression are frequently comorbid, but less is known about the direction of association. According to self-medication theory,^8^ people with depression often use alcohol to cope with negative emotions. Some evidence suggests that during adolescence, depressive symptoms are associated with subsequent increases in alcohol use.^9^ However, adolescent alcohol use might also precede depressive symptoms. Alcohol use might lead to adverse social, psychological, and physical outcomes that increase the risk of subsequent depression, but the potential influence of different patterns of alcohol use on the risk of depression remains unclear.

Theory and evidence support a distinction between alcohol consumption (frequency and quantity of use) and problem use or alcohol use disorders. Various definitions and categorisations of problem use exist, but alcohol dependence is the most severe, rates of which start to increase around age 16 years.^10^ Compared with other excessive drinking behaviours, alcohol dependence could be particularly important as a risk factor for depression. In addition to physical, psychological, and social consequences, alcohol dependence could affect monoamine reward pathways that potentially contribute to addiction and depression.^11^ Conversely, high frequency and quantity consumption of alcohol might not increase the risk of depression because they are often associated with social contact and reflect social norms. Alcohol dependence, but not high frequency or quantity of consumption, might therefore increase the risk of subsequent depression, which would have implications for public health interventions.

**Research in context**

**Evidence before this study**

We searched PubMed, the American Psychological Association, PsycInfo, PsycArticles, and CINAHL databases for studies published in English from database inception to Nov 30, 2022, that investigated alcohol use and depression in adolescents and young adults, using the approved medical subject headings terms “alcohol” and “depressive disorders, major”. We also manually searched reference lists of identified studies. Previous studies on whether alcohol dependence during adolescence increased the risk of subsequent depression had several limitations, and evidence on the frequency and quantity of alcohol consumption was inconsistent. Most studies were done in New Zealand or the USA, some used lifetime measures of alcohol use, which increases the possibility of recall bias, and few adjusted for pre-existing depression and other potential confounders. Most studies were of people born in the 1970s or early 1980s, but patterns of alcohol use have changed among young people. Findings from more recent studies of young people in the 1990s are inconsistent with earlier findings. Few studies tested the hypothesis that alcohol dependence, but not consumption, during adolescence increases the risk of depression during young adulthood. The effects of alcohol dependence and consumption at different timepoints during adolescence, and the rate by which they increase, is poorly understood but could inform the timing of public health interventions.

**Added value of this study**

In a large population-based sample of young people born in England in 1991–92, we examined change in alcohol dependence and consumption from age 16 years to 23 years—a developmental period when average alcohol use increases rapidly. We found evidence of a positive association between alcohol dependence at 18 years of age and depression at 24 years of age. We found no evidence that frequency or quantity of consumption was associated with depression, although we did not directly compare dependence and consumption.

**Implications of all the available evidence**

Alcohol dependence increases the risk of depression in adolescents, but high frequency and quantity of alcohol consumption in the absence of dependence might not. Preventing alcohol dependence during adolescence, or potentially treating it early, could therefore reduce the risk of future depression in young adults, which could have important public health implications. However, heavy alcohol consumption is likely to precede dependence. High frequency and quantity of alcohol consumption therefore remains important to prevent or reduce during adolescence, especially given its associations with other harms, such as injury and antisocial behaviour. Public health interventions to prevent depression among young people could target subthreshold levels of alcohol dependence, which are likely to involve high frequency and quantity of consumption.

Most studies of alcohol use and depression have been done in adults.^12^ In one systematic review of longitudinal studies, only six of 42 studies were of adolescents.^12^ This review found evidence that alcohol use disorders, including dependence, increased risk of subsequent depression.^12^ However, any association with frequency and quantity of consumption appeared to be due to confounding. Only 50% of studies were rated as high quality, and the findings are unlikely to generalise to adolescents.

Among adolescents, studies of whether alcohol dependence increases risk of subsequent depression have several limitations, and evidence on frequency and quantity of consumption is inconsistent. Several studies have found evidence of positive associations between adolescent alcohol misuse or dependence and subsequent depression.^13^, ^14^ However, all of these studies were conducted in New Zealand or the USA. Some studies used lifetime measures of alcohol misuse or dependence, increasing risk of recall bias.^13^ One systematic review of 16 longitudinal studies during adolescence found that higher frequency and quantity of alcohol consumption were associated with increased risk of depression.^13^ However, the quality of studies was low, with few adjusting for pre-existing depression and potential confounders.^13^

Another limitation of research on alcohol use and depression among adolescents is that most participants were born during the 1970s or early 1980s. Patterns of alcohol use have changed among young people,^4^ and evidence from more contemporary samples would inform future public health policy. Additionally, findings from more recent studies are inconsistent with earlier findings. Two studies of adolescents born in the 1990s^15^, ^16^ found no longitudinal evidence of associations between alcohol consumption, misuse, or dependence and subsequent depression.

Few studies have tested the hypothesis that alcohol dependence, but not consumption, during adolescence increases the risk of depression during young adulthood. One review explored whether severity of alcohol use influenced associations with depression during adolescence or young adulthood.^14^ There was some evidence of stronger associations with alcohol use disorders than with consumption. However, that review was narrative and did not report a quality assessment, although most studies were cross-sectional. There is some evidence that alcohol misuse and dependence are positively associated with depression, whereas high levels of consumption decrease risk.^17^, ^18^, ^19^, ^20^ However, these studies were cross-sectional,^17^ small,^20^ or had minimal adjustment for confounders.^18^ These studies did not investigate dependency, which, as the most severe end of the spectrum, might confound associations with consumption. Effects of alcohol dependence and consumption at different timepoints during adolescence, and the rate by which they increase, are also poorly understood and could have implications for public health interventions.

We aimed to examine change in alcohol dependence from ages 16 to 23 years—a developmental period when average alcohol use increases rapidly. We investigated whether higher levels of alcohol dependence (at age 18 years) or a more rapid increase across a short period (rate of change) were associated with depression at age 24 years, and we investigated alcohol consumption to test the hypothesis that dependence, but not consumption, increases the risk of subsequent depression.

## Methods

### Study design and participants

In this prospective cohort study, we included participants of the Avon Longitudinal Study of Parents and Children (ALSPAC), which examined genetic and environmental determinants of health and development. ALSPAC recruited pregnant women resident in Avon, UK, with delivery dates between April 1, 1991, and Dec 31, 1992. Of 14 541 pregnancies, there were 14 676 foetuses, 14 062 livebirths, and 13 988 children alive at age 1 year. Parents and children were followed up regularly via questionnaire and clinic assessments. Data from 2014 were collected using REDCap electronic data capture tools hosted at University of Bristol (Bristol, UK).^21^ Further details on sample characteristics and methodology have been described previously,^22^, ^23^, ^24^ and detailed information can be found on the study website. Information on all available data can be found in the fully searchable data dictionary. Written informed consent was obtained from all mothers, and ethical approval was obtained from ALSPAC Ethics and Law committee (IRB00003312) and Local Research Ethics Committees. The ethics committee approved questionnaires and clinic testing protocols, including methods of gaining consent.

### Measures

The self-report Alcohol Use Disorders Identification Test (AUDIT) was completed by adolescents at about age 16 years, 18 years, 19 years, 21 years, and 23 years. Data were collected during computer-based assessments within clinics at about age 18 years and via questionnaires at about age 16 years, 19 years, 21 years, and 23 years. The AUDIT has high validity and reliability.^25^ We used the continuous consumption subscale (AUDIT-C), summing responses to the first three items, with a range of 0–12 (where 0 indicates no use and 12 is the highest result for frequency and quantity of consumption). Alcohol dependence was assessed using four AUDIT items (at about age 16 years, 18 years, 19 years, 21 years, and 23 years) supplemented by seven items corresponding to DSM-IV symptoms (at about age 18 years, 21 years, and 23 years only), all with response options of never, less than monthly, monthly, weekly, daily, or almost daily, with a range of 0–28 (where 0 indicates no symptoms of dependence and 28 represents the most severe dependence).

We selected confounders based on existing evidence and theoretical assumptions. To avoid variables that could be on the causal pathway, we selected confounders from the same timepoint as our first alcohol exposure variable or earlier. Confounders included sex, housing tenure, maternal education, maternal depressive symptoms (assessed with the ten-item Edinburgh Postnatal Depression Scale), parents' alcohol use (assessed with a questionnaire sent to mothers and their partners), conduct problems at age 4 years (assessed with the five-item conduct problems scale of the Strengths and Difficulties Questionnaire completed by mothers when children were age 4 years), being bullied (at age 16 years, adolescents answered questions on experiences of being bullied since age 12 years as yes or no), and frequency of smoking cigarettes or cannabis (at age 16 years, adolescents answered questions about the use per week). Parent alcohol use represents any parental problematic alcohol use between birth and child age 11 years. Parental problematic alcohol use was measured on multiple occasions from the child's birth to age 11 years with questionnaires sent to mothers and their partners asking whether problematic alcohol use had occurred since the last assessment. Any positive endorsement from either parent of alcoholism or alcohol problems across the first 11 years of the child's life was coded as positive for parental problematic alcohol use. Further detail on confounders and their measurement is in the appendix (p 1). We adjusted for depressive symptoms at age 16 years (the closest timepoint to the start of alcohol growth curves), using the 13-item Short Mood and Feelings Questionnaire (range 0–26, with higher scores representing more severe symptoms).^26^ We did not adjust for depressive symptoms between age 17 years and 24 years because we assumed that these were part of the causal pathway.

### Outcomes

The primary outcome was depression at age 24 years. We used the Clinical Interview Schedule Revised (CIS-R) at age 24 years. CIS-R is a self-administered computerised clinical assessment that assesses the presence and severity of common mental disorder symptoms during the past week.^27^ The CIS-R generates ICD-10 diagnoses, symptom scores for each common mental disorder, and a total score.^27^ Depressive symptom scores range from 0 to 21, with scores of 11 or more approximating clinically significant depression. We used the binary variable for depression at the age of 24 years because depressive symptoms were highly positively skewed. The CIS-R has high reliability and validity, is freely available, and is widely used in population-based surveys, taking approximately 15 min to complete.^27^ The CIS-R has been translated and validated in other settings.

### Statistical analysis

We estimated quadratic latent growth curves to capture non-linear change in consumption and dependence from the age of 16 years to 23 years. Time was measured in years and centred at age 18 years (chosen because it corresponds to the legal age of drinking in the UK and because we had the full set of dependence items at age 18 years). There was minimal variability in quadratic factors for dependence and consumption; therefore, variance in both models (and covariance with intercept and slope) was constrained to zero.^7^ Fit was evaluated by examining residuals for mean and covariance structure, and the final model was evaluated using summary measures including root mean square error of approximation (RMSEA; with values of ≤0·05 indicating a good fit), comparative fit index (CFI; with values of ≥0·90 indicating a good fit), and standardised root mean residual (SRMR; with values of ≤0·08 indicating a good fit).

**Figure 1:**
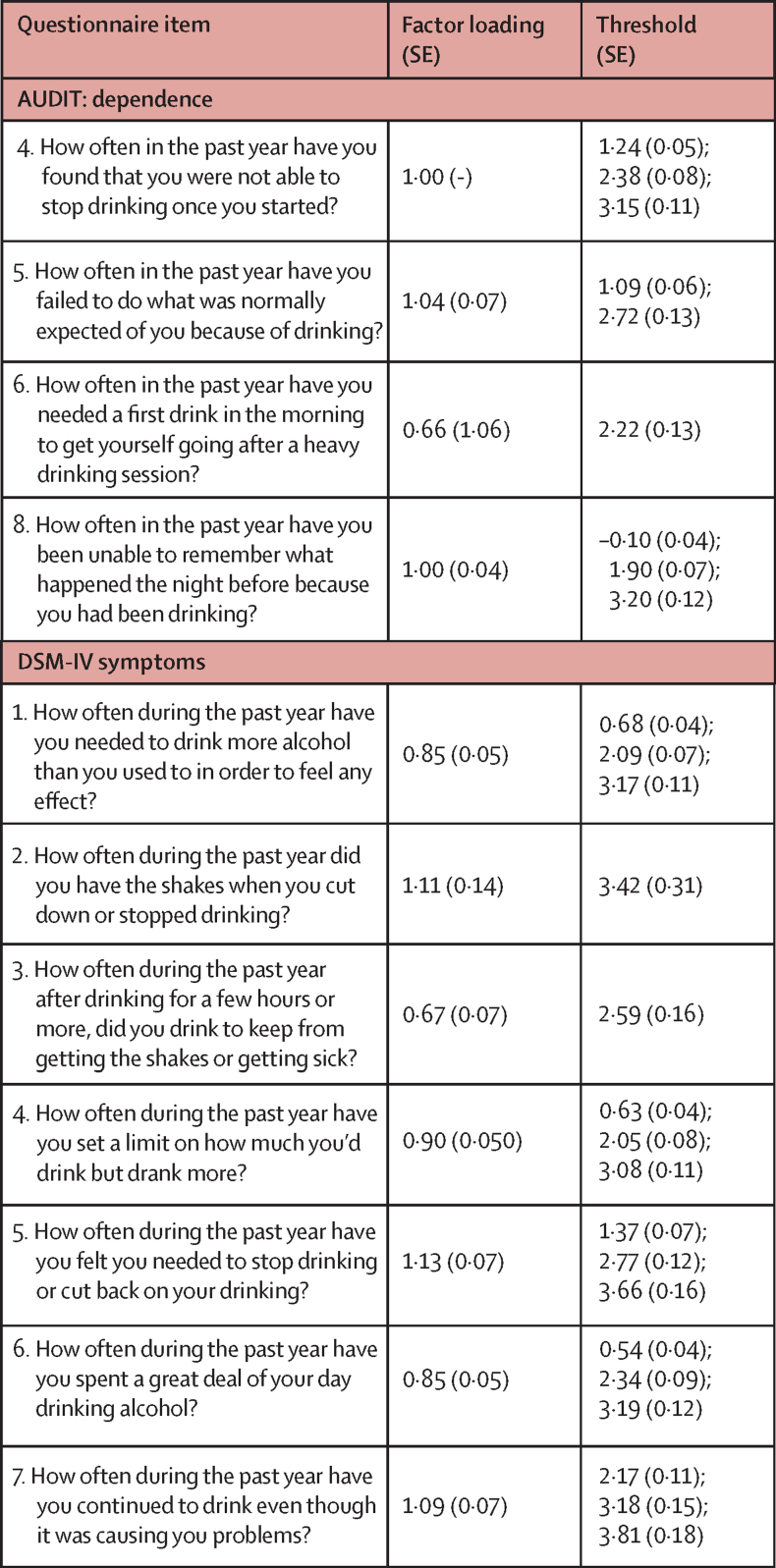
Factor loadings and thresholds (with corresponding SEs) for each dependence item in the fully invariant confirmatory factor analysis AUDIT=Alcohol Use Disorders Identification Test.

For alcohol consumption, we estimated a first-order quadratic latent growth curve using repeated measures of an established scale, AUDIT-C (appendix p 2). For alcohol dependence, given that the seven DSM-IV items were not assessed at every timepoint, dependence was modelled as a latent variable. Ordinal alcohol dependence items at each timepoint were used as indicators for an underlying latent variable, and change was modelled using a second-order quadratic latent growth curve to allow us to include additional items at age 18 years, 21 years, and 23 years.^28^ The mean of the latent intercept was fixed to zero to identify the model. A fully invariant confirmatory factor analysis (loadings and thresholds for dependence items fixed to equality over time) was performed using steps outlined in Mplus (version 7.1), Language Addendum.^29^ Summary fit indices indicated that a fully invariant model was acceptable (RMSEA=0·02, CFI=0·97, and SRMR=0·07); therefore, longitudinal measurement non-invariance was not examined further (figure 1; appendix p 3).

Associations between alcohol growth factors and depression were examined in separate models for consumption and dependence. First, we examined unadjusted associations between alcohol consumption or dependence at age 18 years (latent intercept) and depression at age 24 years, and the association between rate of change in alcohol consumption or dependence per year (linear slope) with depression, adjusting for the latent intercept (at age 18 years). Next, we adjusted for potential confounders by regressing the outcome (depression) on the confounders alongside the exposure (alcohol latent intercept). Effect estimates for associations between alcohol growth factors (latent intercept and linear slope) with depression diagnosis at age 24 years are unstandardised probit coefficients with 95% CIs. Further detail on interpreting probit coefficients is provided in the appendix (p 4).

For the latent growth curve for alcohol consumption, missing data were handled using full information maximum likelihood estimation with robust SEs computed using the observed information matrix.^30^ For the second-order growth model for alcohol dependence, maximum likelihood estimation would have incurred multiple dimensions of integration and been computationally untenable. Weighted least-squares means and variance adjusted (WLSMV) estimation was used to accommodate multiple ordinal latent variable indicators. WLSMV makes the more restrictive assumption of covariate dependent missingness (data are missing completely at random conditional on exogenous variables in the analysis). This assumption was made more plausible by including auxiliary variables in adjusted models.

The analysis sample included participants with data from at least one timepoint for alcohol use and confounders. Further detail on missing data, including the correlates of being missing from the analysis sample, is provided in the appendix (pp 15–16). All analyses were performed using inverse probability weighting^31^ to address potential bias from missing data (appendix p 20). Sensitivity analyses are described in the appendix (p 36) and included regressing depression on the latent intercept fixed at each age of the growth curve; additionally adjusting for household income, crowding, social class, and ethnicity; and rerunning analyses using those with complete data.

All analyses were performed in Mplus (version 8.7). An annotated Mplus script for the adjusted association between alcohol dependence at age 18 years (latent intercept) and depression is provided in the appendix (pp 7–13). Because of using Mplus, p values are provided to 2 significant figures, capped at 3 decimal places, unless p values are less than 0·001.

### Role of the funding source

The funder of the study had no role in study design; in the collection, analysis, and interpretation of data; in the writing of the report; or in the decision to submit the paper for publication.

## Results

We included 3902 adolescents born between April 1, 1991, and Dec 31, 1992, in all analyses: all 3902 participants had complete data on potential confounders and 2222 had data on the primary outcome (appendix p 15). Of 3853 participants with ethnicity data, 3727 (96·7%) were White, and 2264 (58·0%) of 3902 participants were female (table 1). Correlations between alcohol consumption (three AUDIT items) and alcohol dependence (four AUDIT items and seven DSM-IV items) at age 18 years are shown in the appendix (p 22).

**Table 1:** Characteristics of the unweighted and weighted sample using the inverse probability weight

		**Unweighted sample (n=3902)**	Weighted sample*
Ethnicity			
	Non-White	126/3853 (3·3%)	3%
	White	3727/3853 (96·7%)	97%
Sex			
	Female	2264 (58·0%)	50%
	Male	1638 (42·0%)	50%
Housing tenure			
	Owned or mortgaged	3378 (86·6%)	78%
	Rented	524 (13·4%)	22%
Maternal education			
	Beyond high school (age >16 years)	1949 (49·9%)	35%
	High school level or below (age ≤16 years)	1953 (50·1%)	65%
Maternal depressive symptoms†		5·49 (4·42)	6·14 (4·65)
Parental problematic alcohol use‡			
	No	3622 (92·8%)	92%
	Yes	280 (7·2%)	8%
Conduct problems at age 4 years§		1·81 (1·34)	1·91 (1·39)
Being bullied at age 16 years¶			
	No	3244 (83·1%)	82%
	Yes	658 (16·9%)	18%
Frequency of smoking cigarettes at age 16 years‖		0·48 (1·16)	0·53 (1·22)
Frequency of smoking cannabis at age 16 years‖		0·15 (0·55)	0·17 (0·61)
Depressive symptoms at age 16 years**		5·79 (5·52)	5·75 (5·59)
Alcohol consumption (AUDIT-C) at age 18 years††		4·59 (2·50)	4·67 (2·53)
Depression at age 24 years‡‡			
	No	1987/2222 (89·4%)	89%
	Yes	235/2222 (10·6%)	11%

Data are n (%) or mean (SD) unless otherwise specified. AUDIT-C= Alcohol Use Disorders Identification Test continuous consumption subscale.

* Absolute numbers are not provided when reporting weighted percentages given that these might not correspond to a whole number.

† Assessed with the ten-item Edinburgh Postnatal Depression Scale; scores range from 0 to 30, with higher scores indicating more severe symptoms (appendix p 1).

‡ Assessed with questionnaires sent to mothers and their partners (appendix p 1).

§ Assessed with the five-item conduct problems scale of the Strengths and Difficulties Questionnaire (appendix p 1) completed by mothers when children were age 4 years (scores ranged from 0 to 10, with higher scores indicating more severe symptoms).

¶ Adolescents answered questions on experiences of being bullied since the age of 12 years (yes or no).

‖ Cigarette and cannabis use (response options: never or not currently; less than once a week; between one and six times a week; more than six times a week; and every day; higher scores indicate greater frequency of use).

** Assessed with the 13-item Short Mood and Feelings Questionnaire (range 0–26, with higher scores indicating more severe symptoms).^26^

†† Assessed with the three-item AUDIT-C subscale, with higher scores indicating more severe symptoms.

‡‡ Assessed with the Clinical Interview Schedule Revised, with scores ranging from 0 to 21, with scores of 11 or more approximating clinically significant depression.^27^

**Figure 2:**
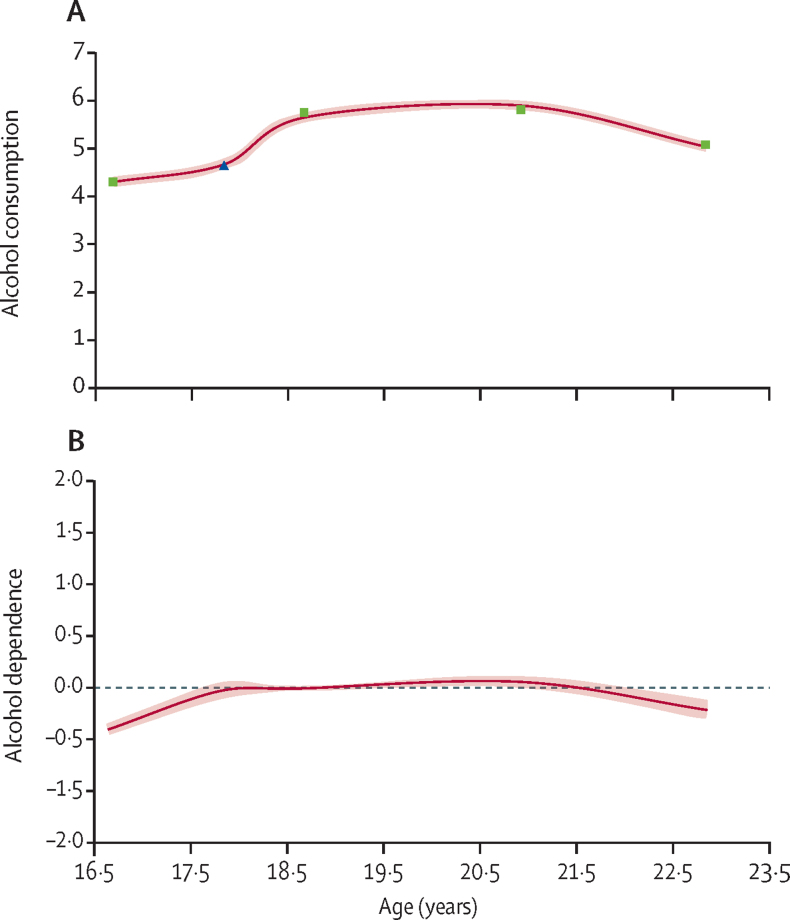
Growth curves showing observed and estimated means for alcohol consumption (A) and estimated means for alcohol dependence (B) for age 16–23 years Shading indicates 95% CIs. Observed and estimated means for alcohol consumption were assessed using the AUDIT-C (range 0–12); squares represent observed means from questionnaire assessments and the triangle represents the observed mean from the computer-based assessment within a clinic. Estimated means for alcohol dependence were assessed using four AUDIT items and seven items corresponding to DSM-IV symptoms and are modelled here as a latent variable with the mean fixed to zero. AUDIT=Alcohol Use Disorders Identification Test. AUDIT-C=Alcohol Use Disorders Identification Test continuous consumption subscale.

The first-order quadratic latent growth model for alcohol consumption provided an acceptable fit (RMSEA=0·06, CFI=0·94, and SRMR=0·07). Using the estimated and observed means for alcohol consumption from age 16 to 23 years, at the start of the growth curve (about age 16·5 years), young people had an average score of 4·3 on AUDIT-C (figure 2A). On average, the highest score of alcohol consumption was 6·0 (at about age 20 years). Means, variances, and correlations between growth factors, and associations between confounders and growth factors are shown in the appendix (p 23).

There was no evidence for an association between alcohol consumption at age 18 years (latent intercept) and depression at age 24 years (probit coefficient 0·01 [95% CI –0·04 to 0·05]; p=0·80; table 2). For the association between rate of change in alcohol consumption per year (linear slope) and depression, the probit coefficient was –0·39 (95% CI –0·79 to 0·02; p=0·061; table 2). After adjustments, there was no evidence for an association between alcohol consumption at age 18 years (latent intercept) and depression at age 24 years (probit coefficient –0·01 [95% CI –0·06 to 0·03]; p=0·60) and no longer evidence for a negative association between rate of change per year (linear slope) and depression (0·01 [–0·40 to 0·42]; p=0·96; table 2; appendix pp 26–27).

**Table 2:** Unadjusted and adjusted associations between growth factors for alcohol consumption and dependence with depression at age 24 years using weighted data

	**Unadjusted analyses**		Adjusted analyses*	
	Unstandardised probit coefficient (95% CI)	p value	Unstandardised probit coefficient (95% CI)	p value
**Alcohol consumption**				
Latent intercept (age 18 years)	0·01 (−0·04 to 0·05)	0·80	−0·01 (−0·06 to 0·03)	0·60
Linear slope†	−0·39 (−0·79 to 0·02)	0·061	0·01 (−0·40 to 0·42)	0·96
**Alcohol dependence**				
Latent intercept (age 18 years)	0·21 (0·11 to 0·32)	<0·001	0·13 (0·02 to 0·25)	0·019
Linear slope†	−0·43 (−1·29 to 0·43)	0·33	0·10 (−0·82 to 1·01)	0·84

* Confounders included sex, housing tenure, maternal education, maternal depressive symptoms, parents' alcohol use, conduct problems at age 4 years, being bullied from age of 12 to 16 years, cannabis and cigarette use at age 16 years, and depressive symptoms at age 16 years.

† All analyses for the linear slope adjust for the latent intercept (at age 18 years).

The second-order quadratic latent growth model for alcohol dependence provided an acceptable fit (RMSEA=0·02, CFI=0·98, and SRMR=0·07). Through the estimated means for alcohol dependence from age 16 to 23 years, at the start of the growth curve, young people had an average score of –0·4 on the latent variable for alcohol dependence (figure 2B). This corresponds to a 4% probability of not being able to stop drinking once started, a 6% probability of failing to do what was normally expected because of drinking, a 1% probability of needing a drink in the morning, and a 36% probability of being unable to remember what happened the night before (appendix pp 5–6). On average, the highest level on the alcohol dependence latent variable was 0·2 (at about age 20 years), which represents an increase in these probabilities to 13%, 17%, 2%, and 61%, respectively. Means, variances, and correlations between growth factors, and associations between confounders and growth factors are shown in the appendix (pp 29, 32).

**Figure 3:**
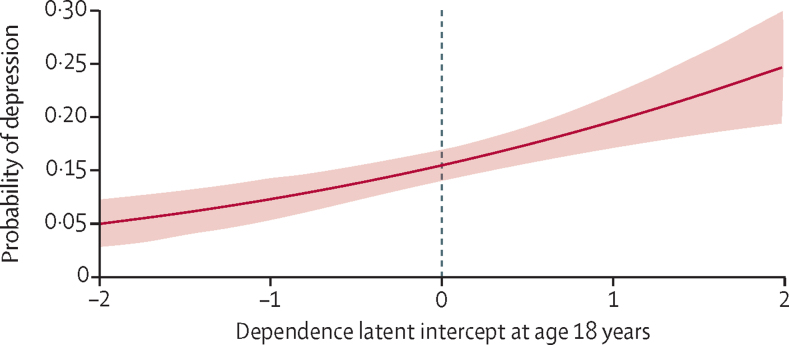
Predicted probability for depression (from the unadjusted model) across the range of alcohol dependence at age 18 years (latent intercept) Shading indicates 95% CIs. Zero indicates mean amount of alcohol dependence.

There was evidence for a positive association between alcohol dependence at age 18 years (latent intercept) and depression at age 24 years (probit coefficient 0·21 [95% CI 0·11 to 0·32]; p<0·001; table 2). Concerning the change in probability of depression as a function of dependence, people with a score of zero on the dependence latent intercept (average score at age 18 years) have an 11% probability of depression (figure 3). This probability increases to 15% for those with a score of 1 on the dependence latent intercept. An increase from zero to 1 on the dependence latent intercept represents a 28% increase in the probability of not being able to stop drinking once started (eg, from 9% to 37%), a 33% increase in the probability of failing to do what was normally expected, a 4% increase in the probability of needing a drink in the morning, and a 34% increase in the probability of being unable to remember what happened the night before (appendix pp 5–6). There was no evidence for an association between rate of change in dependence per year (linear slope) and depression (probit coefficient –0·43 [–1·29 to 0·43]; p=0·33).

After adjustments, there was still evidence for a positive association between alcohol dependence at age 18 years (latent intercept) and depression at age 24 years (probit coefficient 0·13 [95% CI 0·02 to 0·25]; p=0·019; table 2). There was still no evidence for an association between rate of change in dependence per year (linear slope) and depression (probit coefficient 0·10 [–0·82 to 1·01]; p=0.84; table 2; appendix pp 34–35).

We analysed standardised probit coefficients for consumption and dependence at age 18 years (latent intercepts) to compare the magnitude of effect estimates across models. In unadjusted models, 95% CIs for consumption (probit coefficients 0·01 [95% CI –0·08, 0·10]) and dependence (0·21 [0·11 to 0·31]) did not overlap, providing statistical evidence of a difference between effect estimates. In adjusted models, there was a small overlap for consumption (probit coefficients –0·02 [95% CI –0·11 to 0·06]) and dependence (0·11 [0·02 to 0·21]). However, the effect estimate for consumption was close to zero and not within the 95% CI for dependence, and the effect estimate for dependence was not within the 95% CI for consumption. Despite the effect size for consumption being close to zero, we cannot rule out the possibility that effect estimates might not be statistically different, due to the small overlap in 95% CIs, although this seems unlikely. Conclusions from sensitivity analyses were unchanged (appendix pp 36–39). Additionally, when we regressed depression on levels of alcohol consumption or dependence at each age of the growth curve (17–22 years), we found that there was no evidence for an association between alcohol consumption at any age with depression at age 24 years. We also found the association between alcohol dependence and depression was consistent across this developmental period (appendix pp 36–37).

## Discussion

We found evidence that alcohol dependence at age 18 years was associated with depression at age 24 years, which also held when we examined the association between levels of alcohol dependence at each age of the growth curve (17–22 years) and depression at 24 years. We found no evidence that frequency or quantity of consumption was associated with depression, although we did not directly compare dependence and consumption. Our findings therefore suggest that high frequency and quantity of alcohol consumption might not increase the risk of depression during young adulthood, unless there are also features of dependency involved. We found no evidence that a faster increase in levels of dependence across adolescence was associated with depression at age 24 years, and only small variability in rate of change over time. This finding suggests that the magnitude of the association between dependence and depression is fairly constant over adolescence and that the timepoint for considering levels of alcohol dependence (between ages 16 and 23 years) might not be particularly important. Few studies have tested the hypothesis that alcohol dependence, but not consumption, during adolescence increases the risk of depression during young adulthood. We extended previous research using a large contemporary sample, adjusting for a wide range of confounders, and investigating initial levels as well as change in alcohol use over time.

The study also has various limitations. There was substantial attrition in the ALSPAC cohort from birth to age 24 years. Multiple imputation is regarded as optimal for selective attrition but was infeasible given the large number of dependence items that do not occur frequently in young people. We used inverse probability weighting to address bias, and weighted results were similar to the unweighted results. Even when attrition is systematic, biases in ALSPAC are found to be minimal.^32^ Our sample was recruited from one UK region and most participants were White. We were unable to include consumption and dependence in the same model due to high correlations between linear slopes, and 95% CIs overlapped slightly. The possibility that effect estimates might not be statistically different can therefore not be ruled out.

A further limitation is that measures of alcohol consumption and dependence excluded some features of abuse. We did not investigate alcohol abuse without features of dependence, although in DSM-5 alcohol abuse and dependence are brought together into varying degrees of severity of alcohol use disorder.^33^, ^34^ We did not disaggregate frequency from quantity of consumption. Additionally, dependence was measured using up to 11 items and modelled as latent, whereas consumption was measured using three items and modelled as manifest. Using latent variables reduces measurement error; however, it was not possible to model consumption as latent given high correlations between consumption items at age 16 years. This could have resulted in stronger associations for dependence than consumption, but because the association with consumption was negligible, it is unlikely that measurement error completely explained this difference. Our aim was to investigate modifiable risk factors for adolescent depression to inform public health interventions. However, the association between alcohol use and depressive symptoms is likely to be bidirectional. We adjusted for depressive symptoms at age 16 years because these could have contributed to subsequent alcohol problems. We did not include depressive symptoms after age 16 years as a time-varying confounder because the study design (time lags of a year or more between assessments) was inadequate for modelling this interplay. We also did not test modification of associations between alcohol use and depression by sex. We did not have a priori hypotheses about effect modification by sex and statistical tests for interaction are model dependent and often under-powered. We did not formally correct for multiple comparisons because such tests are overly conservative in large samples.

We assessed depression using a binary variable because CIS-R scores are highly positively skewed. Continuous scores capture variation in severity and increase statistical power. We found evidence of an association between alcohol dependence and depression with precise 95% CIs, and it seems unlikely that increased power would alter this finding. The CIS-R assesses depressive symptoms over the past week. Studies with assessments over a longer period, and with longer follow-up times, would be useful. We also did not assess variability over time in the CIS-R. As a final limitation, the possibility of residual confounding can rarely be excluded in observational studies.

Several potential mechanisms could explain our findings. Alcohol dependence is associated with physical, psychological, interpersonal, social, educational, and economic consequences that could lead to depression. Antisocial behaviour and cannabis use could result from alcohol dependence,^7^ and have been found to increase the risk of subsequent depression.^35^

Our findings suggest that preventing alcohol dependence during adolescence, or treating it early, could reduce the risk of depression.^36^ Heavy alcohol consumption is likely to precede dependence. High frequency and quantity of alcohol consumption therefore remain important as targets to prevent or reduce during adolescence, especially given its associations with injury and antisocial behaviour.^37^ Public health interventions to prevent depression could target subthreshold dependence, which is also likely to involve high frequency and quantity of consumption. There is some evidence that psychosocial interventions targeting excessive alcohol use among adolescents in higher education reduced depressive symptoms.^38^ Behavioural interventions such as the Drink Less smartphone app are also being evaluated.^39^ Interventions targeting young people who are dependent or at risk of alcohol dependence could be evaluated and implemented in other settings. Public health messages to prevent depression that are aimed at young people could emphasise dependent aspects of drinking that are harmful. Reducing the frequency and quantity of alcohol consumption during adolescence is also important and could reduce the risk of future dependence and physical health problems. For example, there is evidence that price increases for alcoholic drinks reduce rates of consumption, in the general population and in high-risk groups, such as heavier drinkers and young people.^40^

For the **study website** see http://www.bristol.ac.uk/alspac

For the **data dictionary** see http://www.bris.ac.uk/alspac/researchers/data-access/data-dictionary

For **ALSPAC data** see http://www.bristol.ac.uk/alspac/researchers/access/

For the **list of grant funding** see http://www.bristol.ac.uk/alspac/external/documents/grant-acknowledgements.pdf

## Data sharing

Access to ALSPAC data is through a system of managed access.

**This online publication has been corrected. The corrected version first appeared at thelancet.com/psychiatry on June 9, 2023**

## Declaration of interests

MH has served as Trustee of Society of Study of Addiction and Regional Editor of *Addiction* in the past 3 years. All other authors declare no competing interests.

## References

[1] Mathers CD, Loncar D (2006). Projections of global mortality and burden of disease from 2002 to 2030. PLoS Med.

[2] Kwong ASF, Manley D, Timpson NJ (2019). Identifying critical points of trajectories of depressive symptoms from childhood to young adulthood. J Youth Adolesc.

[3] Bor W, Dean AJ, Najman J, Hayatbakhsh R (2014). Are child and adolescent mental health problems increasing in the 21st century? A systematic review. Aust N Z J Psychiatry.

[4] Vashishtha R, Pennay A, Dietze P, Marzan MB, Room R, Livingston M (2020). Trends in adolescent drinking across 39 high-income countries: exploring the timing and magnitude of decline. Eur J Public Health.

[5] Simons-Morton BG, Farhat T, ter Bogt TFM (2009). Gender specific trends in alcohol use: cross-cultural comparisons from 1998 to 2006 in 24 countries and regions. Int J Public Health.

[6] MacArthur GJ, Smith MC, Melotti R (2012). Patterns of alcohol use and multiple risk behaviour by gender during early and late adolescence: the ALSPAC cohort. J Public Health.

[7] Hammerton G, Mahedy L, Murray J (2017). Effects of excessive alcohol use on antisocial behavior across adolescence and early adulthood. J Am Acad Child Adolesc Psychiatry.

[8] Turner S, Mota N, Bolton J, Sareen J (2018). Self-medication with alcohol or drugs for mood and anxiety disorders: a narrative review of the epidemiological literature. Depress Anxiety.

[9] Edwards AC, Joinson C, Dick DM (2014). The association between depressive symptoms from early to late adolescence and later use and harmful use of alcohol. Eur Child Adolesc Psychiatry.

[10] Hammerton G, Edwards AC, Mahedy L (2020). Externalising pathways to alcohol-related problems in emerging adulthood. J Child Psychol Psychiatry.

[11] Eshel N, Roiser JP (2010). Reward and punishment processing in depression. Biol Psychiatry.

[12] Li J, Wang H, Li M (2020). Effect of alcohol use disorders and alcohol intake on the risk of subsequent depressive symptoms: a systematic review and meta-analysis of cohort studies. Addiction.

[13] Cairns KE, Yap MBH, Pilkington PD, Jorm AF (2014). Risk and protective factors for depression that adolescents can modify: a systematic review and meta-analysis of longitudinal studies. J Affect Disord.

[14] Pedrelli P, Shapero B, Archibald A, Dale C (2016). Alcohol use and depression during adolescence and young adulthood: a summary and interpretation of mixed findings. Curr Addict Rep.

[15] Homman LE, Perra O, Higgins K, O'Neill F (2019). The longitudinal relationship of alcohol problems and depressive symptoms and the impact of externalising symptoms: findings from the Belfast Youth Developmental Study. Soc Psychiatry Psychiatr Epidemiol.

[16] Birrell L, Slade T, Teesson M (2020). Bidirectional relationships in the development of internalising symptoms and alcohol use in adolescence. Drug Alcohol Rev.

[17] Fernandes GS, Lewis G, Hammerton G (2020). Alcohol consumption and internalising disorders in young adults of ALSPAC: a population-based study. J Epidemiol Community Health.

[18] Marmorstein NR (2009). Longitudinal associations between alcohol problems and depressive symptoms: early adolescence through early adulthood. Alcohol Clin Exp Res.

[19] Needham BL (2007). Gender differences in trajectories of depressive symptomatology and substance use during the transition from adolescence to young adulthood. Soc Sci Med.

[20] Mason WA, Kosterman R, Haggerty KP (2008). Dimensions of adolescent alcohol involvement as predictors of young-adult major depression. J Stud Alcohol Drugs.

[21] Harris PA, Taylor R, Thielke R, Payne J, Gonzalez N, Conde JG (2009). Research electronic data capture (REDCap)—a metadata-driven methodology and workflow process for providing translational research informatics support. J Biomed Inform.

[22] Northstone K, Lewcock M, Groom A (2019). The Avon Longitudinal Study of Parents and Children (ALSPAC): an update on the enrolled sample of index children in 2019. Wellcome Open Res.

[23] Boyd A, Golding J, Macleod J (2013). Cohort profile: the ‘children of the 90s’—the index offspring of the Avon Longitudinal Study of Parents and Children. Int J Epidemiol.

[24] Fraser A, Macdonald-Wallis C, Tilling K (2013). Cohort profile: the Avon Longitudinal Study of Parents and Children: ALSPAC mothers cohort. Int J Epidemiol.

[25] Toner P, Böhnke JR, Andersen P, McCambridge J (2019). Alcohol screening and assessment measures for young people: a systematic review and meta-analysis of validation studies. Drug Alcohol Depend.

[26] Angold A, Costello E, Messer S, Pickles A, Winder F, Silver D (1995). The development of a short questionnaire for use in epidemiological studies of depression in children and adolescents. Int J Methods Psychiatr Res.

[27] Lewis G, Pelosi AJ, Araya R, Dunn G (1992). Measuring psychiatric disorder in the community: a standardized assessment for use by lay interviewers. Psychol Med.

[28] Geiser C, Keller BT, Lockhart G (2013). First versus second order latent growth curve models: some insights from latent state-trait theory. Struct Equ Modeling.

[29] Mplus (2013). Version 7.1. Mplus Language Addendum. https://www.statmodel.com/download/Version7.1xLanguage.pdf.

[30] Enders C (2010).

[31] Seaman SR, White IR (2013). Review of inverse probability weighting for dealing with missing data. Stat Methods Med Res.

[32] Wolke D, Waylen A, Samara M (2009). Selective drop-out in longitudinal studies and non-biased prediction of behaviour disorders. Br J Psychiatry.

[33] Gea A, Beunza JJ, Estruch R (2013). Alcohol intake, wine consumption and the development of depression: the PREDIMED study. BMC Med.

[34] Boschloo L, van den Brink W, Penninx BWJH, Wall MM, Hasin DS (2012). Alcohol-use disorder severity predicts first-incidence of depressive disorders. Psychol Med.

[35] Gobbi G, Atkin T, Zytynski T (2019). Association of cannabis use in adolescence and risk of depression, anxiety, and suicidality in young adulthood: a systematic review and meta-analysis. JAMA Psychiatry.

[36] Heron J, MacLeod J, Munafò MR (2012). Patterns of alcohol use in early adolescence predict problem use at age 16. Alcohol Alcohol.

[37] O'Donnell M, Sims S, MacLean MJ, Gonzalez-Izquierdo A, Gilbert R, Stanley FJ (2017). Trends in alcohol-related injury admissions in adolescents in Western Australia and England: population-based cohort study. BMJ Open.

[38] Fredman Stein K, Allen JL, Robinson R, Smith C, Sawyer K, Taylor G (2022). Do interventions principally targeting excessive alcohol use in young people improve depression symptoms?: A systematic review and meta-analysis. BMC Psychiatry.

[39] Garnett C, Oldham M, Angus C (2021). Evaluating the effectiveness of the smartphone app, Drink Less, compared with the NHS alcohol advice webpage, for the reduction of alcohol consumption among hazardous and harmful adult drinkers in the UK at 6-month follow-up: protocol for a randomised controlled trial. Addiction.

[40] Xu X, Chaloupka FJ (2011). The effects of prices on alcohol use and its consequences. Alcohol Res Health.

