# Correction to *Lancet Psychiatry* 2023; 10: 490–98

**DOI:** 10.1016/S2215-0366(23)00198-0

**Published:** 2023-07

*Hammerton G, Lewis G, Heron J, Fernandes G, Hickman M, Lewis G. The association of alcohol dependence and consumption during adolescence with depression in young adulthood, in England: a prospective cohort study.* Lancet Psychiatry *2023; **10:** 490–98*—In this Article, Prof Glyn Lewis's affiliation should have been “Division of Psychiatry, Faculty of Brain Sciences, University College London, London, UK”. This correction has been made to the online version as of June 9, 2023 and will be made to the printed version.

