# Supplementary material for: The association of alcohol dependence and consumption during adolescence with depression in young adulthood, in England: a prospective cohort study
Source: Lancet Psychiatry. 2023 Jul;10(7):490–8. doi: 10.1016/S2215-0366(23)00138-4 (PMC10659986; doi:10.1016/S2215-0366(23)00138-4)
Supplement: Supplementary appendix [file mmc1.pdf]

THE LANCET
Psychiatry

Supplementary appendix

This appendix formed part of the original submission and has been peer reviewed. We post it as supplied by the authors.

Supplement to: Hammerton G, Lewis G, Heron J, Fernandes G, Hickman M, Lewis G. The association of alcohol dependence and consumption during adolescence with depression in young adulthood, in England: a prospective cohort study. *Lancet Psychiatry* 2023; published online June 1. https://doi.org/S2215-0366(23)00138-4.

Supplementary material

Measurement of confounders

Potential confounders were chosen based on evidence from previous studies for an association with both the exposure (alcohol consumption or dependence) and the outcome (depression).[1–11] Child sex was recorded at birth. Maternal questionnaires during pregnancy were used to assess housing tenure (rented versus owned or mortgaged), and maternal education (qualifications at high school level or below versus qualifications beyond high school). Maternal depressive symptoms were assessed at eight weeks postnatal using the 10-item Edinburgh Postnatal Depression Scale (range 0 to 30).[12] Parental problematic alcohol use was assessed on eight occasions from the child's birth to 11 years of age, with questionnaires sent to mothers and their partners, asking whether this had occurred since the last assessment. Any positive endorsement from either parent of problem alcohol use was coded as positive (yes/ no). Conduct problems were assessed using the five-item conduct problems scale of the Strengths and Difficulties Questionnaire[13] completed by mothers when children were four years of age (range 0 to 10). At 16 years of age, adolescents answered questions on experiences of being bullied since the age of 12 (yes/ no), and frequency of cigarette and cannabis use (response options: never/ not currently; less than once a week; between 1 and 6 times a week; more than 6 times a week; every day).

Supplementary Figure 1a. Structural equation model for the latent growth model for alcohol consumption; Alc Con = observed variable measuring alcohol consumption (at age 16, 18, 19, 21 and 23 years); Int=latent intercept growth factor; Slp=linear slope growth factor; Quad=quadratic slope growth factor

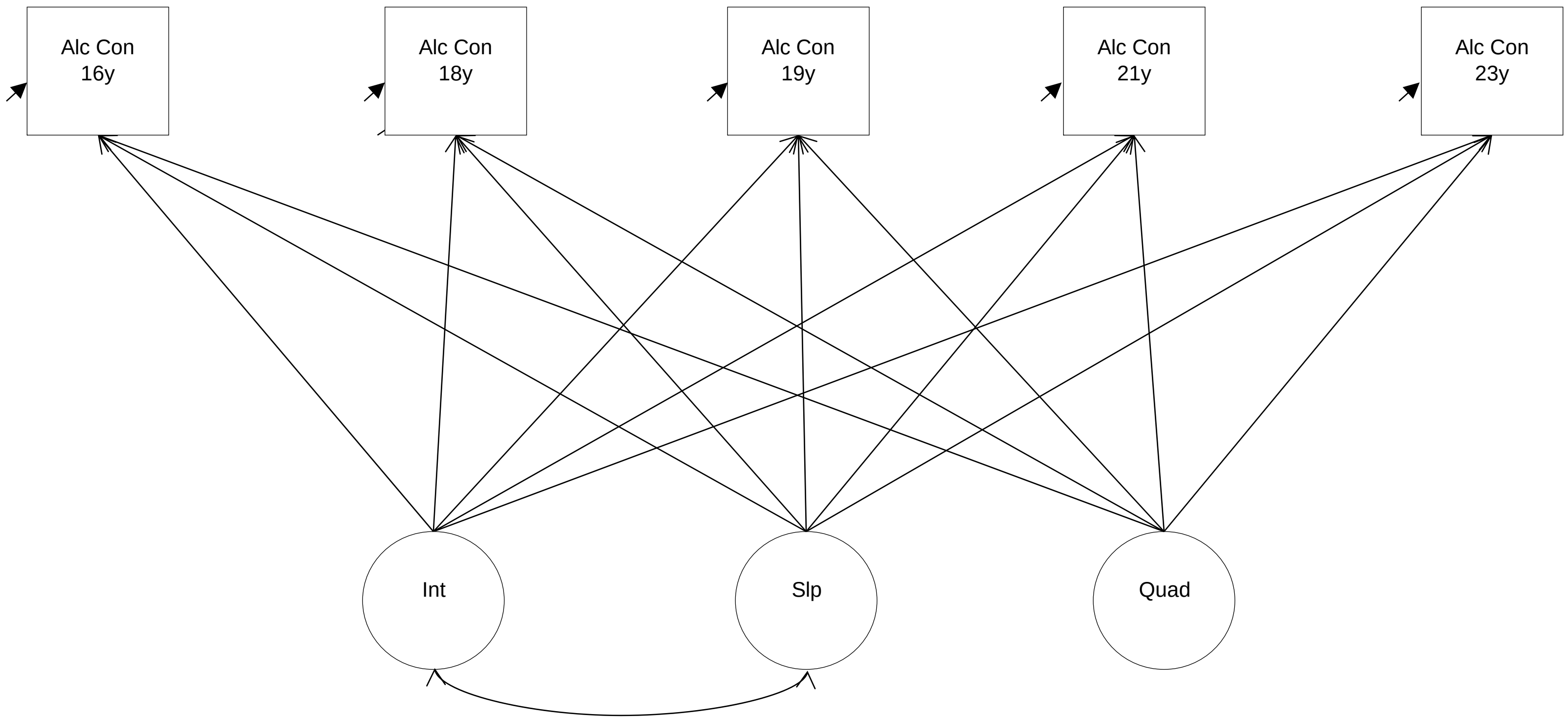

Supplementary Figure 1b. Structural equation model for the second order latent growth model for alcohol dependence; AUD=observed AUDIT dependence item (4, 5, 6 or 8); DSM=observed DSM-IV dependence item (1-7); Alc Dep = latent variable measuring alcohol dependence (at age 16, 18, 19, 21 and 23 years); Int=latent intercept growth factor; Slp=linear slope growth factor; Quad=quadratic slope growth factor; residual covariances were estimated between corresponding items (AUDIT and DSM) over time, but not shown on the figure for clarity

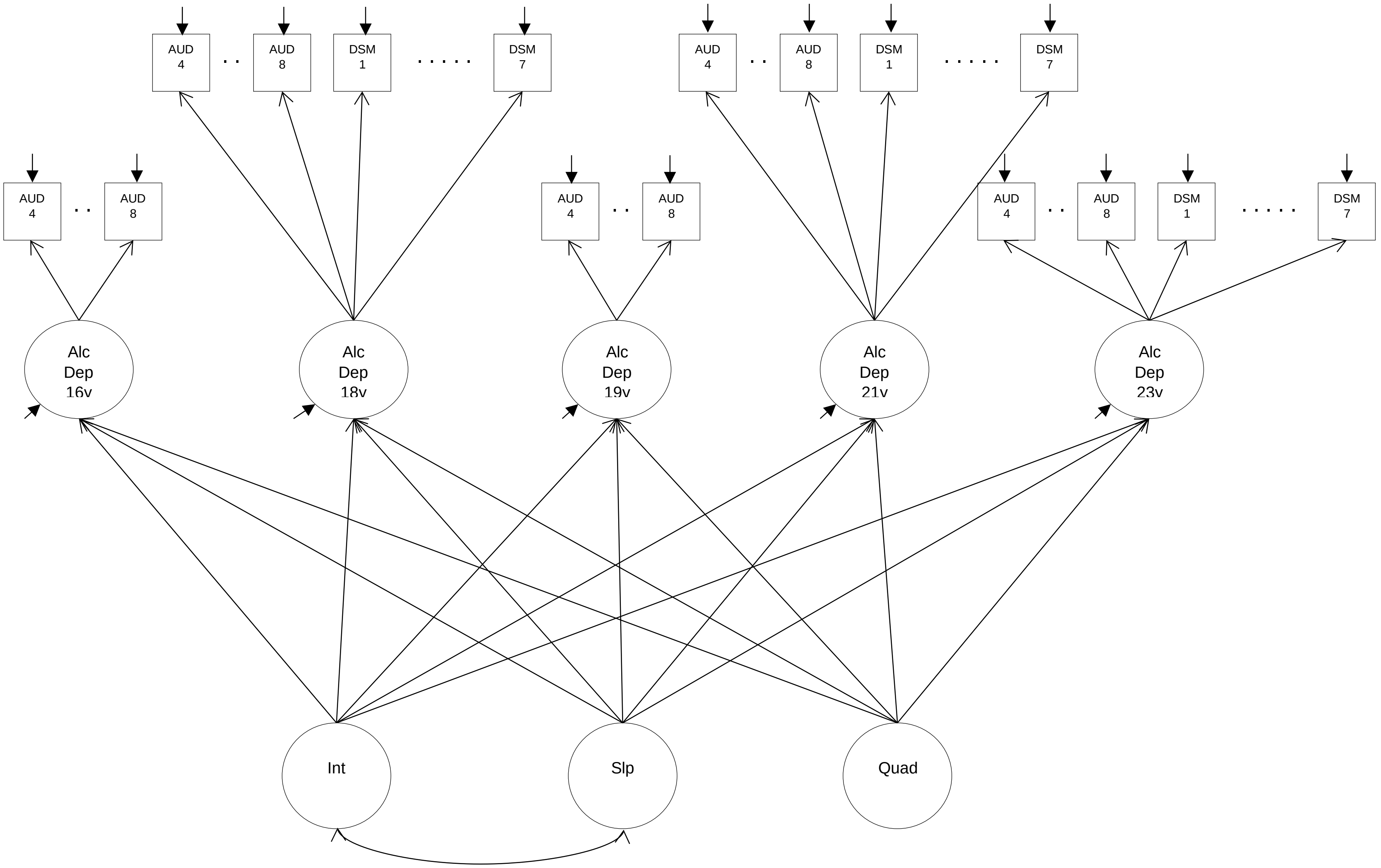

Interpretation of a probit coefficient in the context of the analysis models

Probit coefficients can be interpreted as the difference a unit increase in the exposure has on the predicted z-score for the probability of the outcome. In unadjusted analyses, a probit coefficient of 0.2 indicates that for each unit increase in the exposure, there is a 0.2 increase in the z-score for the probability of depression. A positive sign means the probability of the outcome is increased when the exposure increases; a larger magnitude means this probability increases faster. To facilitate interpretation of effect sizes, we converted z-scores of the probability of depression into probabilities plotted as a function of exposure. For the alcohol consumption growth curve, the scale of the growth factors is equivalent to the scale for the AUDIT-C (range 0 to 12). For the alcohol dependence second order growth curve, the scale of the growth factors is equivalent to the scale of the latent response variable underlying the first ordinal indicator (frequency young person not able to stop drinking once they started). Supplementary Figures 2a and 2b plot the probability of each dependence item being present in the last year across the distribution of the dependence latent intercept at age 18.

Supplementary Figure 2a. Probability of each AUDIT dependence item being reported as present in the last year across the distribution of alcohol dependence at age 18 (latent intercept)

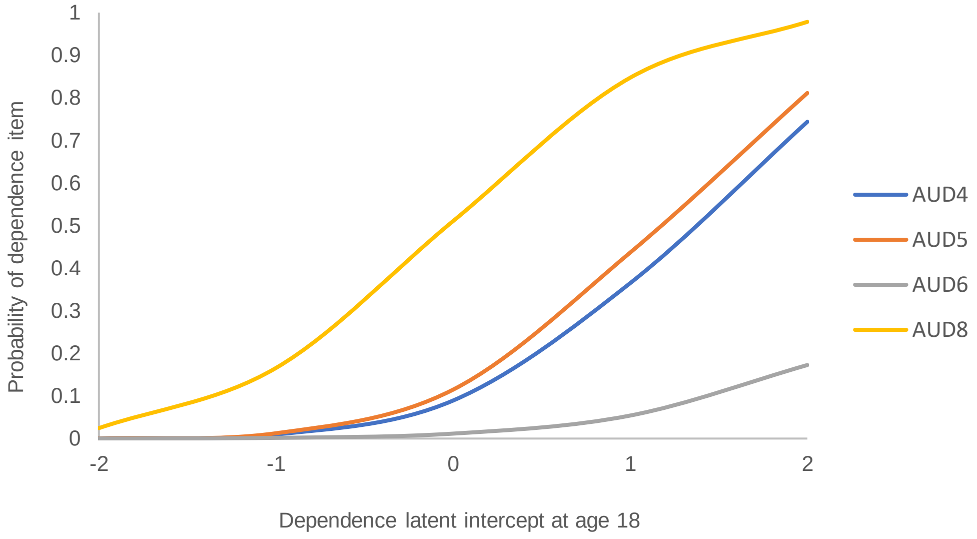

AUD4 = How often in the past year have you found that you were not able to stop drinking once you started? AUD5 = How often in the past year have you failed to do what was normally expected of you because of drinking? AUD6 = How often in the past year have you needed a first drink in the morning to get yourself going after a heavy drinking session? AUD8 = How often in the past year have you been unable to remember what happened the night before because you had been drinking?

Those with a score of zero on alcohol dependence at age 18 (latent intercept representing average levels at age 18 years in the sample) have a 9% probability of not being able to stop drinking once they started, a 11% probability of failing to do what was normally expected of them because of drinking, a 1% probability of needing a first drink in the morning and a 51% probability of being unable to remember what happened the night before because of drinking. A score of one on the latent intercept represents an increase in these probabilities to 37%, 44%, 5% and 85% respectively.

Supplementary Figure 2b. Probability of each DSM-IV dependence item being reported as present in the last year across the distribution of alcohol dependence at age 18 (latent intercept)

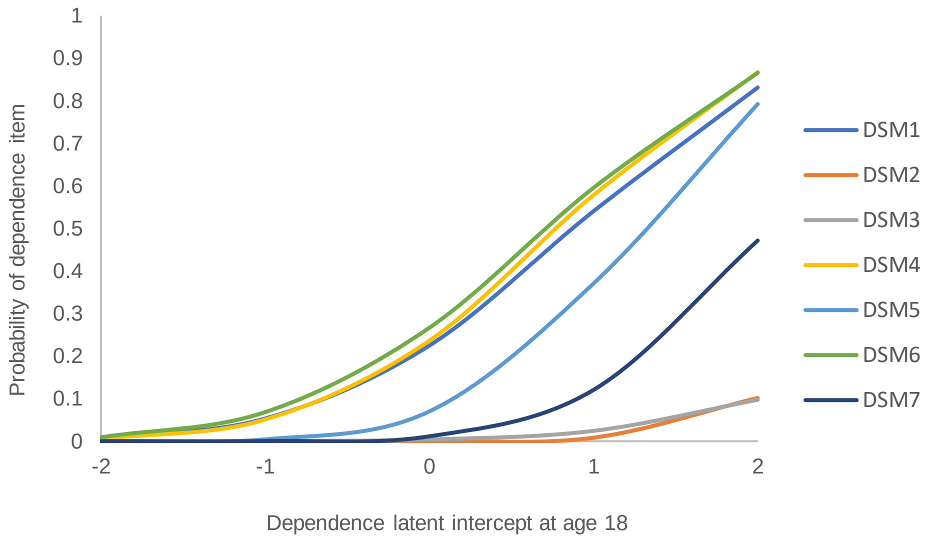

DSM1 = How often during the past year have you needed to drink more alcohol than you used to in order to feel any effect? DSM2 = How often during the past year did you have the shakes when you cut down or stopped drinking? DSM3 = How often during the past year after drinking for a few hours or more, did you drink to keep from getting the shakes or getting sick? DSM4 = How often during the past year have you set a limit on how much you'd drink but drank more? DSM5 = How often during the past year have you felt you needed to stop drinking or cut back on your drinking? DSM6 = How often during the past year have you spent a great deal of your day drinking alcohol? DSM7 = How often during the past year have you continued to drink even though it was causing you problems?

Annotated Mplus input file examining the association between the alcohol dependence latent intercept and depression diagnosis after adjusting for confounders

```
Title: Adjusted association between alcohol dependence latent intercept at
age 18 and depression at age 24

Data: file is dependence.dat;

Variable:

names are

id female tenure mum_edu mum_dep par_alc conduct_4 smoke_16 cannabis_16
bully_16 mfq_16 audc_16 audc_18 audc_19 audc_21 audc_23 aud4_16 aud5_16
aud6_16 aud8_16 aud4_18 aud5_18 aud6_18 aud8_18 aud4_19 aud5_19 aud6_19
aud8_19 aud4_21 aud5_21 aud6_21 aud8_21 aud4_23 aud5_23 aud6_23 aud8_23
dsm1_18 dsm2_18 dsm3_18 dsm4_18 dsm5_18 dsm6_18 dsm7_18 dsm1_21 dsm2_21
dsm3_21 dsm4_21 dsm5_21 dsm6_21 dsm7_21 dsm1_23 dsm2_23 dsm3_23 dsm4_23
dsm5_23 dsm6_23 dsm7_23 dep_24;

! missing values coded as -9999

missing are all (-9999);

usevariables are

! confounders

female tenure mum_edu mum_dep par_alc conduct_4 smoke_16 cannabis_16
bully_16 mfq_16

! alcohol dependence items from AUDIT between age 16 and 23

aud4_16 aud5_16 aud6_16 aud8_16 aud4_18 aud5_18 aud6_18 aud8_18 aud4_19
aud5_19 aud6_19 aud8_19 aud4_21 aud5_21 aud6_21 aud8_21 aud4_23 aud5_23
aud6_23 aud8_23

! alcohol dependence items based on DSM-IV symptoms between age 18 and 23

dsm1_18 dsm2_18 dsm3_18 dsm4_18 dsm5_18 dsm6_18 dsm7_18 dsm1_21 dsm2_21
dsm3_21 dsm4_21 dsm5_21 dsm6_21 dsm7_21 dsm1_23 dsm2_23 dsm3_23 dsm4_23
dsm5_23 dsm6_23 dsm7_23

! depression at age 24 (outcome)

dep_24;

! defining all alcohol dependence items and depression as categorical

categorical are

aud4_16 aud5_16 aud6_16 aud8_16 aud4_18 aud5_18 aud6_18 aud8_18 aud4_19
aud5_19 aud6_19 aud8_19 aud4_21 aud5_21 aud6_21 aud8_21 aud4_23 aud5_23
aud6_23 aud8_23 dsm1_18 dsm2_18 dsm3_18 dsm4_18 dsm5_18 dsm6_18 dsm7_18
dsm1_21 dsm2_21 dsm3_21 dsm4_21 dsm5_21 dsm6_21 dsm7_21 dsm1_23 dsm2_23
dsm3_23 dsm4_23 dsm5_23 dsm6_23 dsm7_23 dep_24;
```

```
! defining sample size as those who have complete data on the alcohol consumption scale (AUDIT-C) from at least one time point

! Mplus will automatically reduce sample size to those with complete data on all the confounders (N = 3,902)

useobservations are

audc_16 NE -9999 OR audc_18 NE -9999 OR audc_19 NE -9999 OR audc_21 NE -9999 OR audc_23 NE -9999;

! defining the inverse probability weight used to address bias from missing data

weight = ipw_weight;

Analysis:

proc = 4(starts);

! using weighted least squares means and variance adjusted (WLSMV) estimation to accommodate multiple ordinal latent variable indicators

estimator = wlsmv;

! using theta parameterisation to work with residual variances rather than scale factors

parameterization = theta;

! including technical 1, 3 and 4 output and confidence intervals

Output: tech1 tech3 tech4 cinterval;

! model specifications

Model:

! first order factors are the alcohol dependence latent variables: dep16, dep18, dep19, dep21, dep23

! second order factors are the growth factors from a quadratic latent growth curve model: latent intercept (i), linear slope (s), quadratic factor (q)

! DEFINING THE FIRST ORDER FACTORS

! using a fully invariant model with loadings and thresholds for dependence items fixed to equality over time using the steps outlined in Mplus version 7.1 (https://www.statmodel.com/download/Version7.1xLanguage.pdf)

! factor loadings for the same latent variable indicator are fixed to equality across time points (e.g., factor loading for aud4 fixed to be the same at ages 16, 18, 19, 21, and 23)

! latent variable for alcohol dependence at age 16 with indicators based on AUDIT questions

dep16 by aud4_16 (aud4)

         aud5_16 (aud5)

         aud6_16 (aud6)

         aud8_16 (aud8);
```

```
! latent variable for alcohol dependence at age 18 with indicators based on AUDIT questions and DSM-IV symptoms

dep18 by aud4_18 (aud4)

         aud5_18 (aud5)

         aud6_18 (aud6)

         aud8_18 (aud8)

         dsm1_18 (dsm1)

         dsm2_18 (dsm2)

         dsm3_18 (dsm3)

         dsm4_18 (dsm4)

         dsm5_18 (dsm5)

         dsm6_18 (dsm6)

         dsm7_18 (dsm7);

! latent variable for alcohol dependence at age 19 with indicators based on AUDIT questions

dep19 by aud4_19 (aud4)

         aud5_19 (aud5)

         aud6_19 (aud6)

         aud8_19 (aud8);

! latent variable for alcohol dependence at age 21 with indicators based on AUDIT questions and DSM-IV symptoms

dep21 by aud4_21 (aud4)

         aud5_21 (aud5)

         aud6_21 (aud6)

         aud8_21 (aud8)

         dsm1_21 (dsm1)

         dsm2_21 (dsm2)

         dsm3_21 (dsm3)

         dsm4_21 (dsm4)

         dsm5_21 (dsm5)

         dsm6_21 (dsm6)

         dsm7_21 (dsm7);

! latent variable for alcohol dependence at age 23 with indicators based on AUDIT questions and DSM-IV symptoms

dep23 by aud4_23 (aud4)

         aud5_23 (aud5)
```

```
        aud6_23 (aud6)
        aud8_23 (aud8)
        dsm1_23 (dsm1)
        dsm2_23 (dsm2)
        dsm3_23 (dsm3)
        dsm4_23 (dsm4)
        dsm5_23 (dsm5)
        dsm6_23 (dsm6)
        dsm7_23 (dsm7);
! thresholds for the same latent variable indicator are fixed to equality across time points (e.g., threshold 1 for aud4 fixed to be the same at ages 16, 18, 19, 21, and 23)
! number of thresholds for each latent variable indicator depends on the number of categories (i.e., an indicator with 4 categories will have 3 thresholds)
! fixing thresholds for indicators based on AUDIT questions
[aud4_16$1 aud4_18$1 aud4_19$1 aud4_21$1 aud4_23$1] (aud4_th1);
[aud4_16$2 aud4_18$2 aud4_19$2 aud4_21$2 aud4_23$2] (aud4_th2);
[aud4_16$3 aud4_18$3 aud4_19$3 aud4_21$3 aud4_23$3] (aud4_th3);
[aud5_16$1 aud5_18$1 aud5_19$1 aud5_21$1 aud5_23$1] (aud5_th1);
[aud5_16$2 aud5_18$2 aud5_19$2 aud5_21$2 aud5_23$2] (aud5_th2);
[aud6_16$1 aud6_18$1 aud6_19$1 aud6_21$1 aud6_23$1] (aud6_th1);
[aud8_16$1 aud4_18$1 aud8_19$1 aud8_21$1 aud8_23$1] (aud8_th1);
[aud8_16$2 aud4_18$2 aud8_19$2 aud8_21$2 aud8_23$2] (aud8_th2);
[aud8_16$3 aud4_18$3 aud8_19$3 aud8_21$3 aud8_23$3] (aud8_th3);
! fixing thresholds for indicators based on DSM-IV symptoms
[dsm1_18$1 dsm1_21$1 dsm1_23$1] (dsm1_th1);
[dsm1_18$2 dsm1_21$2 dsm1_23$2] (dsm1_th2);
[dsm1_18$3 dsm1_21$3 dsm1_23$3] (dsm1_th3);
[dsm2_18$1 dsm2_21$1 dsm2_23$1] (dsm2_th1);
[dsm3_18$1 dsm3_21$1 dsm3_23$1] (dsm3_th1);
[dsm4_18$1 dsm4_21$1 dsm4_23$1] (dsm4_th1);
[dsm4_18$2 dsm4_21$2 dsm4_23$2] (dsm4_th2);
[dsm4_18$3 dsm4_21$3 dsm4_23$3] (dsm4_th3);
[dsm5_18$1 dsm5_21$1 dsm5_23$1] (dsm5_th1);
[dsm5_18$2 dsm5_21$2 dsm5_23$2] (dsm5_th2);
```

```
[dsm5_18$3 dsm5_21$3 dsm5_23$3] (dsm5_th3);

[dsm6_18$1 dsm6_21$1 dsm6_23$1] (dsm6_th1);

[dsm6_18$2 dsm6_21$2 dsm6_23$2] (dsm6_th2);

[dsm6_18$3 dsm6_21$3 dsm6_23$3] (dsm6_th3);

[dsm7_18$1 dsm7_21$1 dsm7_23$1] (dsm7_th1);

[dsm7_18$2 dsm7_21$2 dsm7_23$2] (dsm7_th2);

[dsm7_18$3 dsm7_21$3 dsm7_23$3] (dsm7_th3);

! residual variances for indicators based on AUDIT questions at age 16
freely estimated

aud4_16 aud5_16 aud6_16 aud8_16;

! residual variances for indicators based on AUDIT questions and DSM-IV
symptoms at age 18 set to 1

aud4_18@1 aud5_18@1 aud6_18@1 aud8_18@1 dsm1_18@1 dsm2_18@1 dsm3_18@1
dsm4_18@1 dsm5_18@1 dsm6_18@1 dsm7_18@1;

! residual variances for indicators based on AUDIT questions at age 19
freely estimated

aud4_19 aud5_19 aud6_19 aud8_19;

! residual variances for indicators based on AUDIT questions and DSM-IV
symptoms at age 21 freely estimated

aud4_21 aud5_21 aud6_21 aud8_21 dsm1_21 dsm2_21 dsm3_21 dsm4_21 dsm5_21
dsm6_21 dsm7_21;

! residual variances for indicators based on AUDIT questions and DSM-IV
symptoms at age 23 freely estimated

aud4_23 aud5_23 aud6_23 aud8_23 dsm1_23 dsm2_23 dsm3_23 dsm4_23 dsm5_23
dsm6_23 dsm7_23;

! residual covariances freely estimated between the same latent class
indicator across time points

! residual covariances for indicators based on AUDIT questions

aud4_16 with aud4_18 aud4_19 aud4_21 aud4_23;

aud4_18 with aud4_19 aud4_21 aud4_23;

aud4_19 with aud4_21 aud4_23;

aud4_21 with aud4_23;

aud5_16 with aud5_18 aud5_19 aud5_21 aud5_23;

aud5_18 with aud5_19 aud5_21 aud5_23;

aud5_19 with aud5_21 aud5_23;

aud5_21 with aud5_23;

aud6_16 with aud6_18 aud6_19 aud6_21 aud6_23;

aud6_18 with aud6_19 aud6_21 aud6_23;
```

```
aud6_19 with aud6_21 aud6_23;

aud6_21 with aud6_23;

aud8_16 with aud8_18 aud8_19 aud8_21 aud8_23;

aud8_18 with aud8_19 aud8_21 aud8_23;

aud8_19 with aud8_21 aud8_23;

aud8_21 with aud8_23;

! residual covariances for indicators based on DSM-IV symptoms

dsm1_18 with dsm1_21 dsm1_23;

dsm1_21 with dsm1_23;

dsm2_18 with dsm2_21 dsm2_23;

dsm2_21 with dsm2_23;

dsm3_18 with dsm3_21 dsm3_23;

dsm3_21 with dsm3_23;

dsm4_18 with dsm4_21 dsm4_23;

dsm4_21 with dsm4_23;

dsm5_18 with dsm5_21 dsm5_23;

dsm5_21 with dsm5_23;

dsm6_18 with dsm6_21 dsm6_23;

dsm6_21 with dsm6_23;

dsm7_18 with dsm7_21 dsm7_23;

dsm7_21 with dsm7_23;

! SETTING UP THE SECOND-ORDER GROWTH MODEL

! estimating a quadratic latent growth curve using alcohol dependence
latent variables (first order factors) from age 16 to 23 with the latent
intercept fixed at age 18

i s q | dep16@-1.32 dep18@-0.17 dep19@0.66 dep21@2.91 dep23@4.83;

! estimating variance of the latent intercept and linear slope (second
order factors)

! variance of quadratic slope set to zero given minimal variability

i s q@0;

! estimating covariance between latent intercept and linear slope

! covariances for quadratic slope will be automatically set to zero

i with s;

! estimating mean of linear slope and quadratic slope

! mean of latent intercept is fixed to zero to identify the model

[i@0 s q];
```

```
! covariance between first order factors is set to zero given that their
relationship is explained by the second order factors (latent intercept,
linear slope and quadratic factor)

dep16 with dep18@0 dep19@0 dep21@0 dep23@0;

dep18 with dep19@0 dep21@0 dep23@0;

dep19 with dep21@0 dep23@0;

dep21 with dep23@0;

! covariance between first order and second order factors set to zero

! covariances for quadratic slope will be automatically set to zero

dep16 dep18 dep19 dep21 dep23 with i@0 s@0;

! first order factors are centred (mean set to zero)

[dep16@0 dep19@0 dep21@0 dep23@0];

! additional parameter included for first order factor assessed during a
clinic (rather than questionnaire) to allow trajectory function to absorb
artefactual differences between clinic and questionnaire data

[dep18];

! time-specific residual variances for first-order factors permitted to be
heteroskedastic between but not within assessment technique

dep16 dep19 dep21 dep23 (same);

dep18;

! PROBIT REGRESSION MODEL FOR THE ASSOCIATION BETWEEN THE LATENT INTERCEPT
FOR ALCOHOL DEPENDENCE AND DEPRESSION

! regressing depression on alcohol latent intercept and confounders

dep_24 on i female tenure mum_edu mum_dep par_alc conduct_4 smoke_16
cannabis_16 bully_16 mfq_16;
```

Supplementary Figure 3. Flow chart of retention in the Avon Longitudinal Study of Parents and Children (ALSPAC) sample

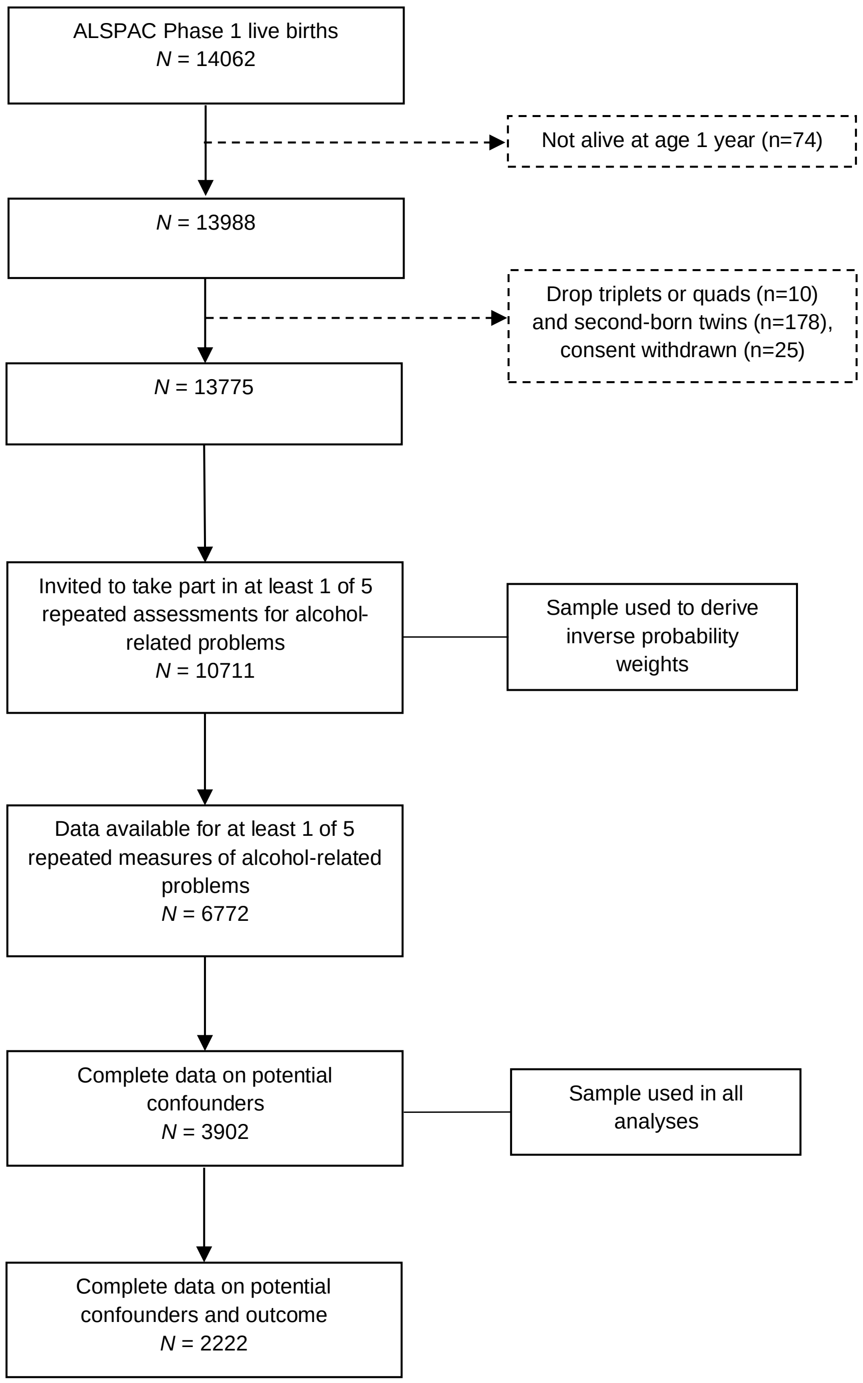

Detail on the pattern and correlates of missing data

The analysis sample included those with data from at least one time-point for alcohol use and confounders (*N* = 3902). Of this sample, 1034 (27%) had complete data on alcohol consumption at all five time-points, 1057 (27%) had complete data at four time-points, 768 (20%) had complete data at three time points, 603 (15%) had complete data at two time-points, and 440 (11%) had complete data at one time point. Supplementary Table 2a shows the percentage of missing data and associations with being missing from the analysis sample for key study variables. Supplementary Table 2a shows that confounders included in the analysis models (including sex, housing tenure, maternal education, maternal depression, child conduct problems, frequency of smoking, and depression symptoms at age 16) were strongly associated with missingness.

Supplementary Table 2b shows the percentage of missing data and associations with missingness for an additional selection of demographic variables. Maternal questionnaires completed during pregnancy or in the early postnatal period were used to assess household crowding (up to one person per room in house (1pprm) or more than one person per room (1+pprm)), ethnicity (non-white or white), parity (study child 1st, 2nd, 3rd or subsequent child born in family), mother smoked in pregnancy (no or yes), and marital status (married, never married or widowed/ divorced). Social class was based on the Registrat General's classification of occupations and grouped into four categories, using the highest social class of either parent: I (professional); II (managerial and technical); III (skilled manual or non-manual); and IV (semi-skilled manual) or V (unskilled). For household income, the average was taken from assessments when the study child was aged 33 months and aged 47 months and split into quintiles. This measure was rescaled to account for estimated housing

benefits, family size and composition, and was expressed in 1995 prices. Supplementary Table 2b shows that all demographic variables were strongly associated with missingness.

Supplementary Table 1. Question wording, response categories, and how the items were analysed for all alcohol items

Questionnaire Item	Response options	Analysis
**AUDIT-consumption**		
1. How often do you have a drink containing alcohol?	0=never; 1=monthly or less; 2=2-4 times a month; 3=2-3 times a week; 4=4 or more times a week	Numeric variable derived at ages 16, 18, 19, 21 and 23 by summing responses to these three items (range 0-12)
2. How many units containing alcohol do you have on a typical day when you are drinking?	0=0 to 2; 1=3 or 4; 2=5 or 6; 3=7 to 9; 4=10 or more	
3. How often do you have six or more units on one occasion?	0=never; 1=less than monthly; 2=monthly; 3=weekly; 4=daily or almost daily	
**AUDIT-dependence:** How often in the past year have you…		
4. …found that you were not able to stop drinking once you started?	0=never; 1=less than monthly; 2=monthly; 3=weekly*; 4=daily or almost daily*	Ordinal (or binary) latent variable indictors at ages 16, 18, 19, 21 and 23
5. …failed to do what was normally expected of you because of drinking?	0=never; 1=less than monthly; 2=monthly*; 3=weekly*; 4=daily or almost daily*	
6. …needed a first drink in the morning to get yourself going after a heavy drinking session?	0=never; 1=less than monthly*; 2=monthly*; 3=weekly*; 4=daily or almost daily*	
8. …been unable to remember what happened the night before because you had been drinking?	0=never; 1=less than monthly; 2=monthly; 3=weekly*; 4=daily or almost daily*	
**DSM-IV symptoms:** How often during the past year…		
1. …have you needed to drink more alcohol than you used to in order to feel any effect?	0=never; 1=less than monthly; 2=monthly; 3=weekly*; 4=daily or almost daily*	Ordinal (or binary) latent variable indictors at ages 18, 21 and 23
2. …did you have the shakes when you cut down or stopped drinking?	0=never; 1=less than monthly*; 2=monthly*; 3=weekly*; 4=daily or almost daily*	
3. …after drinking for a few hours or more, did you drink to keep from getting the shakes or getting sick?		
4. …have you set a limit on how much you'd drink but drank more?	0=never; 1=less than monthly; 2=monthly; 3=weekly*; 4=daily or almost daily*	
5. …have you felt you needed to stop drinking or cut back on your drinking?		
6. …have you spent a great deal of your day drinking alcohol?		
7. …have you continued to drink even though it was causing you problems?		

* these response options were collapsed together to prevent cell sizes less than 10

Supplementary Table 2a. Percentage of missing data and associations with being missing from the analysis sample for key study variables

	**Full cohort (*N* = 13775)**	**Analysis sample (*N* = 3902)**	**Missing from analysis sample (*N* = 9873)**	**Analysis sample (*N* = 3902)**	
**Variable**	n (%) missing	n (%) missing	n (%) or Mean (SD)	n (%) or Mean (SD)	OR (95% CI)
**Confounders**					
Sex	0	0			p < 0.001
Male	-	-	5466 (55%)	1638 (42%)	Reference
Female	-	-	4407 (45%)	2264 (58%)	0.58 (0.54, 0.63)
Housing tenure	951 (7%)	0			p < 0.001
Owned or mortgaged	-	-	6019 (67%)	3378 (87%)	Reference
Rented	-	-	2903 (33%)	524 (13%)	3.11 (2.81, 3.44)
Maternal education	1557 (11%)	0			p < 0.001
Beyond high school	-	-	2369 (28%)	1949 (50%)	Reference
High school or below	-	-	5947 (72%)	1953 (50%)	2.51 (2.32, 2.71)
Maternal depressive symptoms	2234 (16%)	0	6.34 (4.94)	5.49 (4.42)	1.04 (1.03, 1.05); p< 0.001
Parental problem alcohol use	1689 (12%)	0			p = 0.54
No	-	-	7571 (93%)	3622 (93%)	Reference
Yes	-	-	613 (7%)	280 (7%)	1.05 (0.90, 1.21)
Conduct problems, age 4	4418 (32%)	0	2.08 (1.46)	1.81 (1.34)	1.15 (1.11, 1.18); p< 0.001
Being bullied, age 16	8989 (65%)	0			p = 0.046
No	-	-	710 (80%)	3244 (83%)	Reference
Yes	-	-	174 (20%)	658 (17%)	1.21 (1.00, 1.45)
Frequency of smoking cigarettes, age 16	8983 (65%)	0	0.61 (1.28)	0.48 (1.16)	1.09 (1.03, 1.15); p =0.0056
Frequency of smoking cannabis, age 16	8984 (65%)	0	0.16 (0.54)	0.15 (0.55)	1.00 (0.88, 1.15); p = 0.95
Depressive symptoms, age 16	9051 (66%)	0	6.36 (6.04)	5.79 (5.52)	1.02 (1.00, 1.03); p= 0.0093
**Exposures**					
Alcohol consumption, age 16	9107 (66%)	94 (2%)	4.17 (3.01)	4.25 (2.83)	0.99 (0.96, 1.01); p = 0.35
Alcohol consumption, age 18	9889 (72%)	1465 (38%)	4.87 (2.65)	4.59 (2.50)	1.04 (1.02, 1.07); p= 0.0010
Alcohol consumption, age 19	10659 (77%)	1653 (42%)	5.47 (3.00)	5.61 (2.76)	0.98 (0.96, 1.01); p= 0.21
Alcohol consumption, age 21	9880 (72%)	1397 (36%)	5.64 (2.75)	5.81 (2.69)	0.98 (0.95, 1.00); p= 0.069
Alcohol consumption, age 23	10128 (74%)	1553 (40%)	4.91 (2.65)	5.03 (2.50)	0.98 (0.96, 1.01); p= 0.19
**Outcome**					
Depression, age 24	10145 (74%)	1680 (43%)			p = 0.50
No	-	-	1249 (89%)	1987 (89%)	Reference
Yes	-	-	159 (11%)	235 (11%)	1.08 (0.87, 1.33)

Supplementary Table 2b. Percentage of missing data and associations with being missing from the analysis sample for demographic variables and indicators for the inverse probability weight (IPW)

	**Full cohort (*N* = 13775)**	**Analysis sample (*N* = 3902)**	**Missing from analysis sample (*N* = 9873)**	**Analysis sample (*N* = 3902)**	
**Variable**	n (%) missing	n (%) missing	n (%)	n (%)	OR (95% CI)
**Demographics**					
Income	3994 (29%)	157 (4%)			p < 0.001
Highest 20%	-	-	988 (16%)	997 (27%)	Reference
3	-	-	1065 (18%)	888 (24%)	1.21 (1.07, 1.37)
2	-	-	1205 (20%)	740 (20%)	1.64 (1.45, 1.87)
1	-	-	1283 (21%)	656 (18%)	1.97 (1.74, 2.24)
Lowest 20%	-	-	1495 (25%)	464 (12%)	3.25 (2.83, 3.73)
Social class	2461 (18%)	138 (4%)			p < 0.001
I	-	-	750 (10%)	755 (20%)	Reference
II	-	-	2975 (39%)	1747 (46%)	1.71 (1.52, 1.93)
III	-	-	3262 (43%)	1153 (31%)	2.85 (2.52, 3.22)
IV or V	-	-	563 (7%)	109 (3%)	5.20 (4.14, 6.54)
Household crowding	1172 (9%)	49 (1%)			p < 0.001
Up to 1pprm	-	-	7997 (91%)	3738 (97%)	Reference
1+ pprm	-	-	753 (9%)	115 (3%)	3.06 (2.51, 3.74)
Ethnicity	1869 (14%)	49 (1%)			p < 0.001
Non-white	-	-	474 (6%)	126 (3%)	Reference
White	-	-	7579 (94%)	3727 (97%)	0.54 (0.44, 0.66)
**IPW indicators**					
Maternal smoking in pregnancy	804 (6%)	11 (0.3%)			p < 0.001
No	-	-	6333 (70%)	3285 (84%)	Reference
Yes	-	-	2747 (30%)	606 (16%)	2.35 (2.13, 2.59)
Marital status	872 (6%)	11 (0.3%)			p < 0.001
Married	-	-	6418 (71%)	3249 (83%)	Reference
Never married	-	-	1999 (22%)	463 (12%)	2.19 (1.96, 2.44)
Widowed/ divorced	-	-	595 (7%)	179 (5%)	1.68 (1.42, 2.00)
Parity	1052 (8%)	36 (1%)			p < 0.001
First	-	-	3806 (43%)	1893 (49%)	Reference
Second	-	-	3089 (35%)	1362 (35%)	1.13 (1.04, 1.23)
Third	-	-	1349 (15%)	467 (12%)	1.44 (1.28, 1.62)
Fourth plus	-	-	613 (7%)	144 (4%)	2.12 (1.75, 2.56)

Detail on the inverse probability weighting used to address missing data

Inverse probability weighting (IPW) has been recommended over alternative methods for dealing with missing data (such as multiple imputation) in situations where whole blocks of data are missing for a large proportion of individuals [14]. Weights were derived from a logistic regression analysis between a set of measures assessed in pregnancy or early postnatal period that were independently predictive of missing data and variables in the analysis (maternal education, maternal smoking in pregnancy, maternal depression symptoms, marital status, parity, housing tenure and child sex) and inclusion in the final sample (N = 3902/10,711). Minimal missing data on indicators used to derive weights were singly imputed as the modal or mean value (all indicators had < 8% of values missing apart from maternal depression symptoms which had 11% missing). Maternal depression symptoms were log transformed to address the skewed distribution. The Hosmer-Lemeshow test was used assess the fit of the missingness model, with results showing no indication of poor fit (Hosmer-Lemeshow χ^2 (df) = 9.66 (8); p = 0.29). Weights ranged from 1.4 to 12.5.

All analyses were run using inverse probability weighting to address any potential bias caused by participant drop-out. Weighted estimates were similar to unweighted estimates - see Supplementary Table 3 below for a comparison of associations for alcohol dependence and alcohol consumption after adjusting for potential confounders.

Supplementary Table 3. Comparison of adjusted associations between growth factors for alcohol consumption and dependence with depression at age 24 years using weighted and unweighted data; *N* = 3902

	Weighted analyses	Unweighted analyses
Alcohol consumption		
Latent intercept (18 years)	-0.01 (-0.06, 0.03); p=0.60	-0.01 (-0.06, 0.03); p=0.57
Linear slope	0.01 (-0.40, 0.42); p=0.96	-0.11 (-0.52, 0.30); p=0.59
Alcohol dependence		
Latent intercept (18 years)	0.13 (0.02, 0.25); p=0.019	0.12 (0.02, 0.23); p=0.023
Linear slope	0.10 (-0.82, 1.01); p=0.84	0.09 (-0.86, 1.04); p=0.85

Supplementary Table 4. Correlation matrix for all alcohol consumption and dependence items at age 18 years; *N* = 2439

	1.	2.	3.	4.	5.	6.	7.	8.	9.	10.	11.	12.	13.	14.
1. AUDIT1-C	1													
2. AUDIT2-C	0.34 (0.02)	1												
3. AUDIT3-C	0.72 (0.01)	0.68 (0.01)	1											
4. AUDIT4-D	0.42 (0.03)	0.38 (0.03)	0.53 (0.02)	1										
5. AUDIT5-D	0.41 (0.03)	0.34 (0.03)	0.49 (0.03)	0.62 (0.03)	1									
6. AUDIT6-D	0.34 (0.05)	0.32 (0.05)	0.38 (0.05)	0.57 (0.05)	0.53 (0.05)	1								
7. AUDIT8-D	0.49 (0.02)	0.50 (0.02)	0.66 (0.02)	0.58 (0.02)	0.58 (0.03)	0.43 (0.05)	1							
8. DSM1	0.49 (0.03)	0.44 (0.03)	0.61 (0.02)	0.52 (0.03)	0.52 (0.03)	0.54 (0.05)	0.56 (0.02)	1						
9. DSM2	0.40 (0.05)	0.36 (0.06)	0.53 (0.04)	0.69 (0.04)	0.55 (0.06)	0.53 (0.08)	0.58 (0.05)	0.59 (0.05)	1					
10. DSM3	0.27 (0.06)	0.33 (0.06)	0.41 (0.05)	0.50 (0.05)	0.46 (0.06)	0.53 (0.08)	0.44 (0.05)	0.46 (0.05)	0.75 (0.06)	1				
11. DSM4	0.44 (0.03)	0.36 (0.03)	0.52 (0.02)	0.65 (0.02)	0.60 (0.03)	0.43 (0.05)	0.57 (0.02)	0.53 (0.02)	0.53 (0.06)	0.43 (0.05)	1			
12. DSM5	0.47 (0.03)	0.32 (0.03)	0.53 (0.03)	0.64 (0.03)	0.67 (0.03)	0.57 (0.05)	0.56 (0.03)	0.56 (0.03)	0.61 (0.05)	0.49 (0.06)	0.65 (0.02)	1		
13. DSM6	0.56 (0.02)	0.49 (0.02)	0.66 (0.02)	0.60 (0.03)	0.46 (0.03)	0.57 (0.05)	0.56 (0.02)	0.56 (0.03)	0.56 (0.05)	0.55 (0.05)	0.54 (0.03)	0.56 (0.03)	1	
14. DSM7	0.39 (0.04)	0.36 (0.04)	0.50 (0.04)	0.65 (0.03)	0.66 (0.03)	0.55 (0.05)	0.58 (0.03)	0.62 (0.03)	0.64 (0.06)	0.60 (0.05)	0.55 (0.03)	0.63 (0.03)	0.57 (0.03)	1

AUDIT1-C: alcohol frequency, AUDIT2-C: typical consumption, AUDIT3-C: bingeing, AUDIT4-D: unable to stop, AUDIT5-D: failed to do what was expected, AUDIT6-D: needed drink in morning, AUDIT8-D: unable to remember, DSM1: tolerance, DSM2: withdrawal (shakes), DSM3: withdrawal (shakes and sick), DSM4: drank more than limit, DSM5: need to cut down, DSM6: time, DSM7: causing problems

Detail on the first order latent growth curve for alcohol consumption

A first order quadratic latent growth curve was estimated to capture non-linear change in alcohol consumption from age 16 to 23 years. Means, variances, and correlations for the alcohol dependence growth factors in the unconditional model are shown in Supplementary Table 5 below.

Supplementary Table 5. Means, variances, and correlations for alcohol consumption growth factors; showing parameter estimate (standard error); *N* = 3902

	1.	2.	3.
1. Latent intercept (at age 18 years)	1		
2. Linear slope	-0.35 (0.03)	1	
3. Quadratic factor	0	0	0
Mean	5.31 (0.05)	0.59 (0.02)	-0.13 (0.01)
Variance	4.51 (0.13)	0.12 (0.01)	0

The latent intercept for the alcohol consumption growth curve was fixed at 18 years of age with an average of 5.31 points (standard error (SE) = 0.05) on the AUDIT-C scale, and initially increased 0.59 points per year (SE = 0.02), before decreasing with an average quadratic factor of -0.13 (SE = 0.01). With the AUDIT-C, ≥5 is a validated cut-point representing hazardous alcohol use.[15] There was a negative correlation between the latent intercept and linear slope suggesting that those who have lower levels of alcohol consumption at age 18, increase more rapidly over time (e.g., individual trajectories show a fanning in pattern, with variability in alcohol consumption decreasing over time).

Multivariable associations between potential confounders and alcohol consumption at age 18 (latent intercept) and the rate of change in alcohol consumption per year (linear slope) are shown in Supplementary Table 6 below. Female sex had a negative association with levels of alcohol consumption at age 18 years (latent intercept) and the rate of change per year (linear slope). This suggests that females have lower levels of alcohol consumption at age 18 compared to males, and increase more slowly over time. The pattern of findings was similar for low maternal education. Parental problematic alcohol use was positively associated with alcohol consumption at age 18 (latent intercept), but showed no association with rate of change per year (linear slope). Again, getting bullied (and living in rented accommodation) were associated with lower levels of alcohol consumption at age 18 (latent intercept), but showed no association with rate of change per year (linear slope). Finally, frequency of smoking cigarettes/ cannabis and depression symptoms were associated with higher levels of consumption at age 18 (latent intercept) and a slower rate of change per year (linear slope).

Supplementary Table 6. Multivariable associations between potential confounders and alcohol consumption growth factors; showing unstandardised beta coefficients (95% confidence interval); *N* = 3902

Potential confounders	Alcohol consumption growth factors	
	Latent intercept (age 18)	Linear slope
Sex		
Male	Reference	
Female	-0.33 (-0.49, -0.17); p<0.001	-0.05 (-0.09, -0.01); p=0.018
Housing tenure		
Owned or mortgaged	Reference	
Rented	-0.28 (-0.52, -0.05); p=0.018	-0.05 (-0.11, 0.01); p=0.092
Maternal education		
Beyond high school	Reference	
High school level or below	-0.20 (-0.35, -0.06); p=0.010	-0.06 (-0.09, -0.02); p=0.002
Maternal depressive symptoms	0.00 (-0.02, 0.02); p=0.83	0.00 (-0.01, 0.004); p=0.89
Parental problematic alcohol use		
No	Reference	
Yes	0.48 (0.15, 0.81); p=0.010	0.02 (-0.07, 0.11); p=0.64
Conduct problems, age 4	-0.03 (-0.09, 0.03); p=0.30	-0.02 (-0.03, -0.002); p=0.026
Being bullied, age 16		
No	Reference	
Yes	-0.45 (-0.65, -0.24); p<0.001	-0.01 (-0.06, 0.05); p=0.73
Frequency of smoking cigarettes, age 16	0.54 (0.46, 0.62); p<0.001	-0.09 (-0.12, -0.07); p<0.001
Frequency of smoking cannabis, age 16	0.56 (0.39, 0.73); p<0.001	-0.05 (-0.10, -0.001); p=0.047
Depressive symptoms, age 16	0.01 (-0.001, 0.03); p=0.076	-0.01 (-0.01, -0.001); p=0.014

Given a very small variance (0.010; SE = 0.002) for the quadratic factor, it was constrained to zero in the main analyses. This resulted in a very small change in the model fit (quadratic variance estimated: RMSEA=0.05; CF1=0.97; SRMR=0.04; quadratic variance zero: RMSEA=0.06; CF1=0.94; SRMR=0.07). As pointed out by a reviewer, the decision to constrain the quadratic factor variance to zero could have affected the results. Therefore, we have re-run the fully adjusted model for alcohol consumption with the quadratic variance freely estimated (Supplementary Table 7).

Supplementary Table 7. Comparison of adjusted associations between growth factors for alcohol consumption with depression at age 24 years using a model where the quadratic variance was constrained to zero (main analyses) and a model where it was freely estimated; *N* = 3902

	Quadratic variance constrained to zero	Quadratic variance freely estimated
Alcohol consumption		
Latent intercept (18 years)	-0.01 (-0.06, 0.03); p=0.60	-0.01 (-0.06, 0.03); p=0.59
Linear slope	0.01 (-0.40, 0.42); p=0.96	0.65 (-1.18, 2.48); p=0.49
Quadratic factor	-	5.96 (-10.50, 22.41); p=0.48

Supplementary Figure 4a. Path model showing association between alcohol consumption at age 18 (latent intercept) and depression after adjusting for confounders; Alc Con = observed variable measuring alcohol consumption (at age 16, 18, 19, 21 and 23 years); Int=latent intercept growth factor; Slp=linear slope growth factor; Quad=quadratic slope growth factor

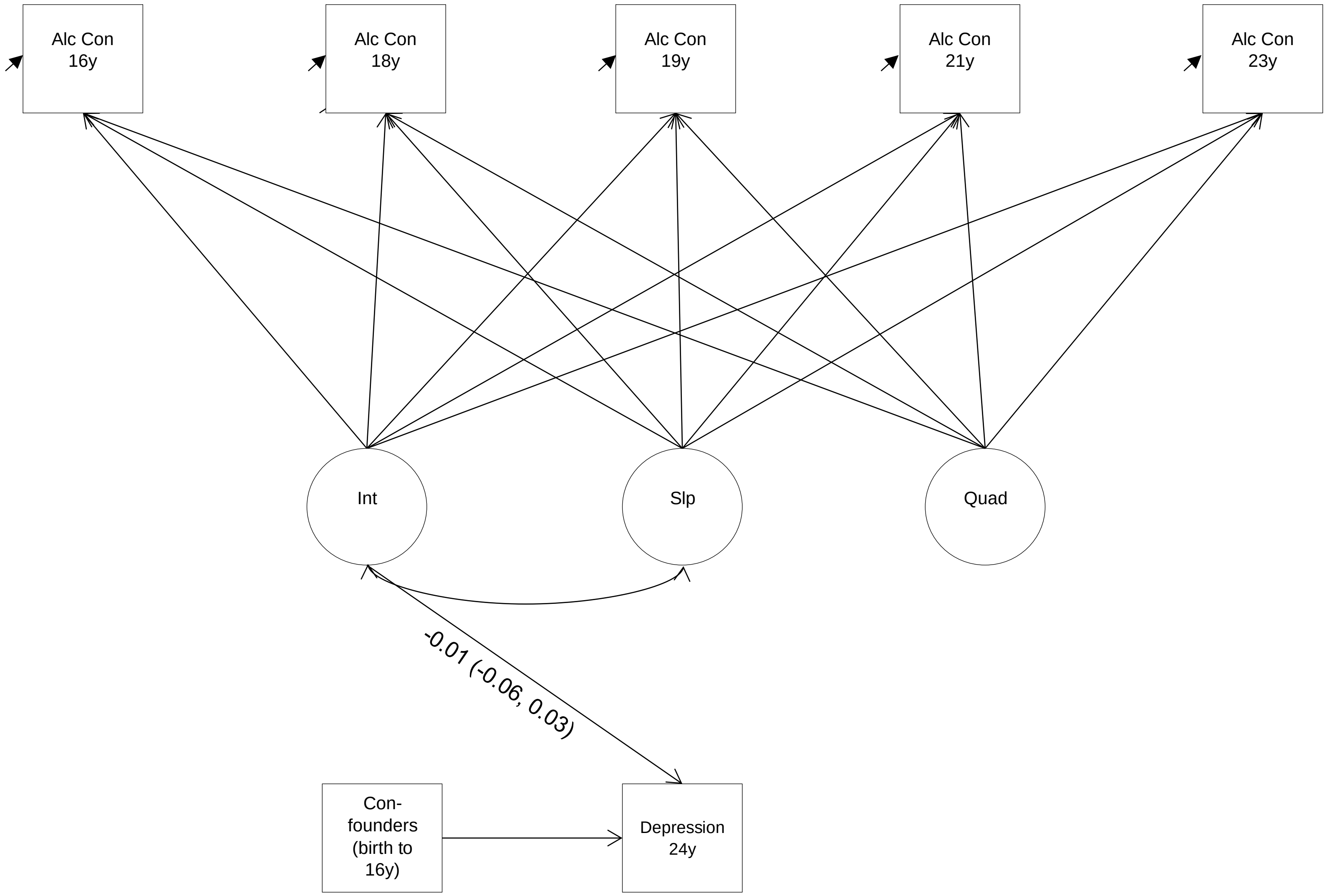

Supplementary Figure 4b. Path model showing association between rate of change in alcohol consumption per year (linear slope) and depression after adjusting for the latent intercept and confounders; Alc Con = observed variable measuring alcohol consumption (at age 16, 18, 19, 21 and 23 years); Int=latent intercept growth factor; Slp=linear slope growth factor; Quad=quadratic slope growth factor

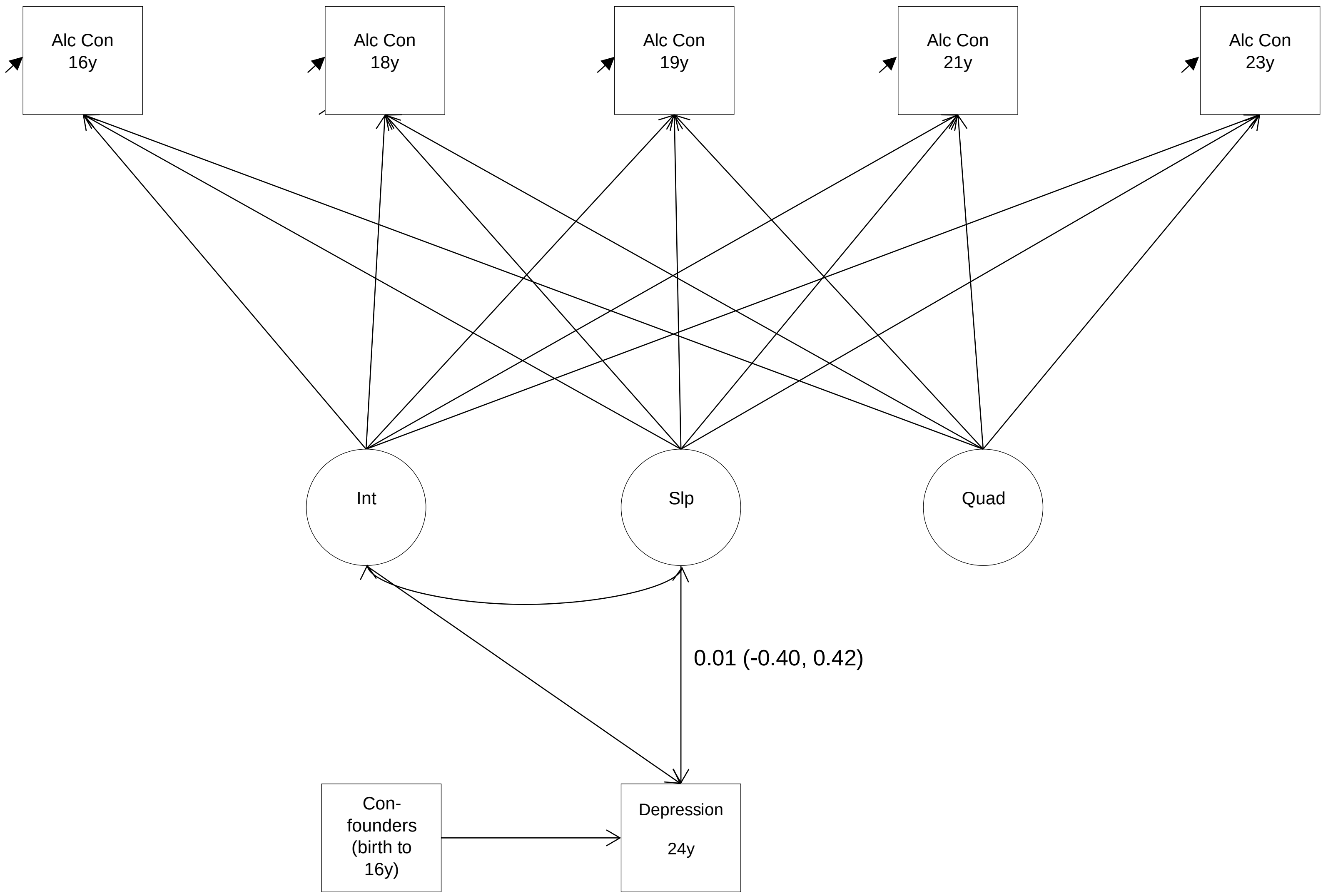

Detail on the second order latent growth curve for alcohol dependence

A second order quadratic latent growth curve was estimated to capture non-linear change in alcohol dependence from age 16 to 23 years. Means, variances, and correlations for the alcohol dependence growth factors in the unconditional model are shown in Supplementary Table 8 below.

Supplementary Table 8. Means, variances, and correlations for alcohol dependence growth factors; showing parameter estimate (standard error); *N* = 3902

	1.	2.	3.
1. Intercept (at age 18 years)	1		
2. Linear slope	-0.34 (0.06)	1	
3. Quadratic factor	0	0	0
Mean	0	0.22 (0.02)	-0.05 (0.01)
Variance	1.01 (0.10)	0.03 (0.004)	0

The mean of the latent intercept (at age 18 years) was fixed to zero to identify the model. Alcohol dependence initially increased 0.22 points on the dependence latent variable per year (SE = 0.02), before decreasing with an average quadratic factor of -0.05 (SE = 0.01). Again, there was a negative correlation between the latent intercept and linear slope suggesting that those who have lower levels of alcohol dependence at age 18, increase more rapidly over time (e.g., individual trajectories show a fanning in pattern, with variability in alcohol dependence decreasing over time). This negative correlation shows the

importance of considering the rate of change in alcohol dependence in addition to average levels, given that those who increase rapidly in levels of dependence across adolescence do not necessarily begin with high levels.

Multivariable associations between potential confounders and alcohol dependence at age 18 (latent intercept) and the rate of change in alcohol dependence per year (linear slope) are shown in Supplementary Table 9 below. Female sex had no association with alcohol dependence at age 18 years (latent intercept) but had a negative association with the rate of change per year (linear slope). This suggests that although males and females don't differ on their levels of alcohol dependence at age 18, females increase more slowly over time. The pattern of findings was similar for low maternal education. In contrast to this, parental problematic alcohol use, frequency of smoking cannabis, and depression symptoms were positively associated with alcohol dependence at age 18 (latent intercept) but showed no association with rate of change per year (linear slope). Somewhat surprisingly, getting bullied between ages 12 and 16 years was associated with lower levels of alcohol dependence at age 18(latent intercept). Finally, frequency of cigarette smoking was associated with higher levels of dependence at age 18 (latent intercept) but a slower rate of change per year (linear slope).

In both latent growth curve models, an additional parameter was included to allow trajectory functions to absorb artefactual differences between clinic and questionnaire data. These differences might be due to the tendency to more readily report alcohol use within questionnaires completed at home.[16] This is equivalent to including an assessment-technique dummy variable as a fixed effect in multilevel modelling formulations of latent

growth models. The time-specific residual variances for repeated measures were permitted to be heteroskedastic between but not within assessment technique.

Supplementary Table 9. Multivariable associations between potential confounders and alcohol dependence growth factors; showing unstandardised beta coefficients (95% confidence interval); *N* = 3902

**Potential confounders**	**Alcohol dependence growth factors**	
	**Latent intercept (age 18)**	**Linear slope**
Sex		
Male	Reference	
Female	-0.02 (-0.12, 0.07); p=0.61	-0.05 (-0.08, -0.03); p<0.001
Housing tenure		
Owned or mortgaged	Reference	
Rented	-0.11 (-0.25, 0.03); p=0.13	-0.02 (-0.06, 0.02); p=0.42
Maternal education		
Beyond high school	Reference	
High school level or below	-0.05 (-0.13, 0.04); p=0.30	-0.05 (-0.08, -0.03); p<0.001
Maternal depressive symptoms	0.01 (-0.001, 0.02); p=0.077	0.000 (-0.003, 0.003); p=0.84
Parental problematic alcohol use		
No	Reference	
Yes	0.22 (0.03, 0.41); p=0.026	0.01 (-0.05, 0.06); p=0.77
Conduct problems, age 4	-0.02 (-0.06, 0.01); p=0.21	0.00 (-0.01, 0.01); p=0.98
Being bullied, age 16		
No	Reference	
Yes	-0.20 (-0.33, -0.08); p=0.002	-0.01 (-0.04, 0.03); p=0.71
Frequency of smoking cigarettes, age 16	0.22 (0.17, 0.27); p<0.001	-0.03 (-0.05, -0.02); p<0.001
Frequency of smoking cannabis, age 16	0.24 (0.13, 0.35); p<0.001	0.01 (-0.02, 0.04); p=0.42
Depressive symptoms, age 16	0.03 (0.02, 0.04); p<0.001	-0.002 (-0.01, 0.000); p=0.11

Given a very small variance (0.002; SE = 0.001) for the quadratic factor, it was constrained to zero in the main analyses. This resulted in a very small improvement in the model fit (quadratic variance estimated: RMSEA=0.02; CF1=0.97; SRMR=0.07; quadratic variance zero: RMSEA=0.02; CF1=0.98; SRMR=0.07). As pointed out by a reviewer, the decision to constrain the quadratic factor variance to zero could have affected the results. Therefore, we have re-run the fully adjusted model for alcohol dependence with the quadratic variance freely estimated (Supplementary Table 10).

Supplementary Table 10. Comparison of adjusted associations between growth factors for alcohol dependence with depression at age 24 years using a model where the quadratic variance was constrained to zero (main analyses) and a model where it was freely estimated; *N* = 3902

	Quadratic variance constrained to zero	Quadratic variance freely estimated
Alcohol dependence		
Latent intercept (18 years)	0.13 (0.02, 0.25); p=0.019	0.13 (0.02, 0.25); p=0.019
Linear slope	0.10 (-0.82, 1.01); p=0.84	0.10 (-0.75, 0.94); p=0.83
Quadratic factor	-	0.34 (-6.51, 7.18); p=0.92

Supplementary Figure 5a. Path model showing association between alcohol dependence at age 18 (latent intercept) and depression after adjusting for confounders; Alc Dep = latent variable measuring alcohol dependence (at age 16, 18, 19, 21 and 23 years); Int=latent intercept growth factor; Slp=linear slope growth factor; Quad=quadratic slope growth factor; observed AUDIT and DSM-IV dependence items not shown on figure for clarity

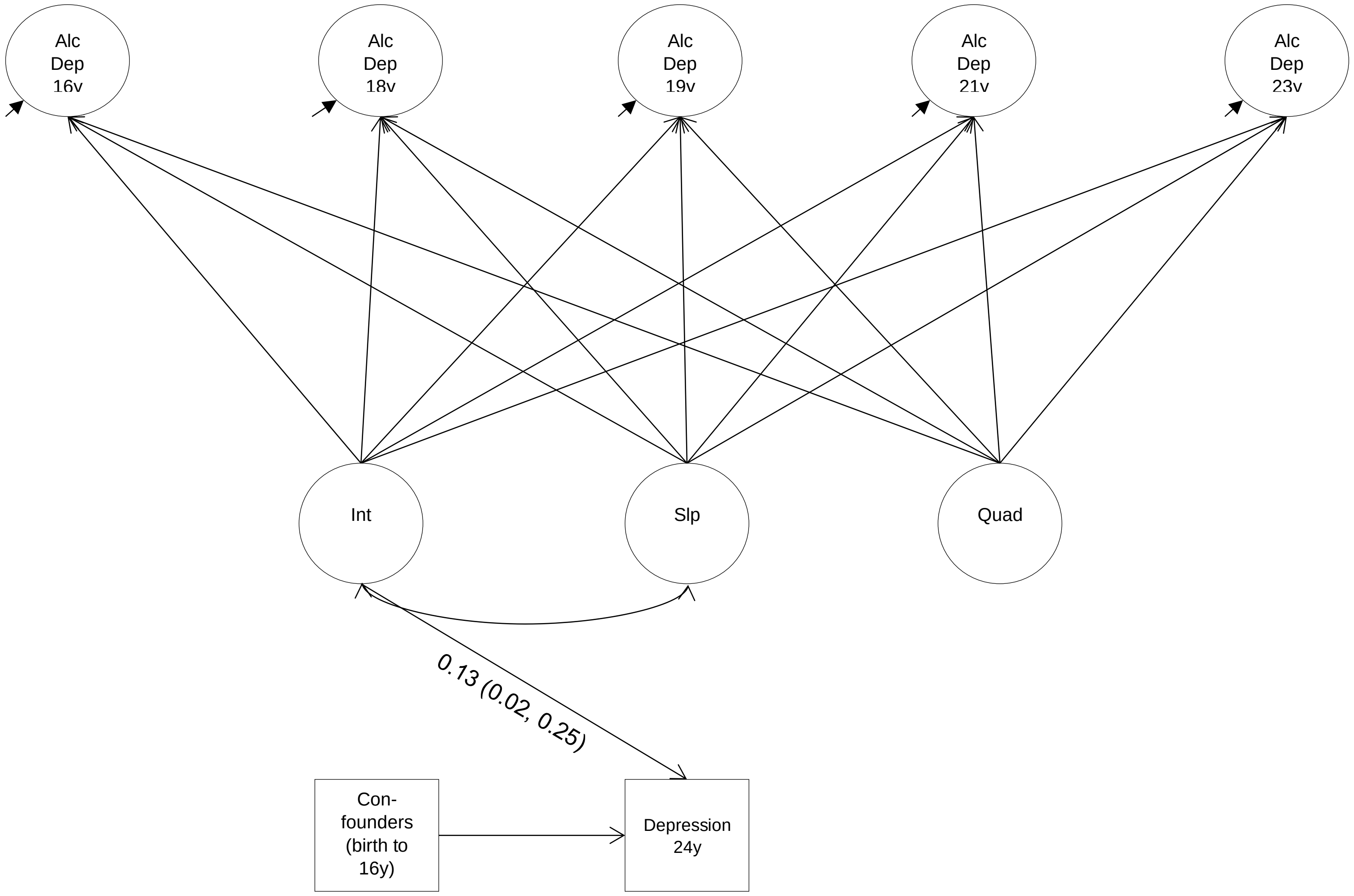

Supplementary Figure 5b. Path model showing association between rate of change in alcohol dependence per year (linear slope) and depression after adjusting for the latent intercept and confounders; Alc Dep = latent variable measuring alcohol dependence (at age 16, 18, 19, 21 and 23 years); Int=latent intercept growth factor; Slp=linear slope growth factor; Quad=quadratic slope growth factor; observed AUDIT and DSM-IV dependence items not shown on figure for clarity

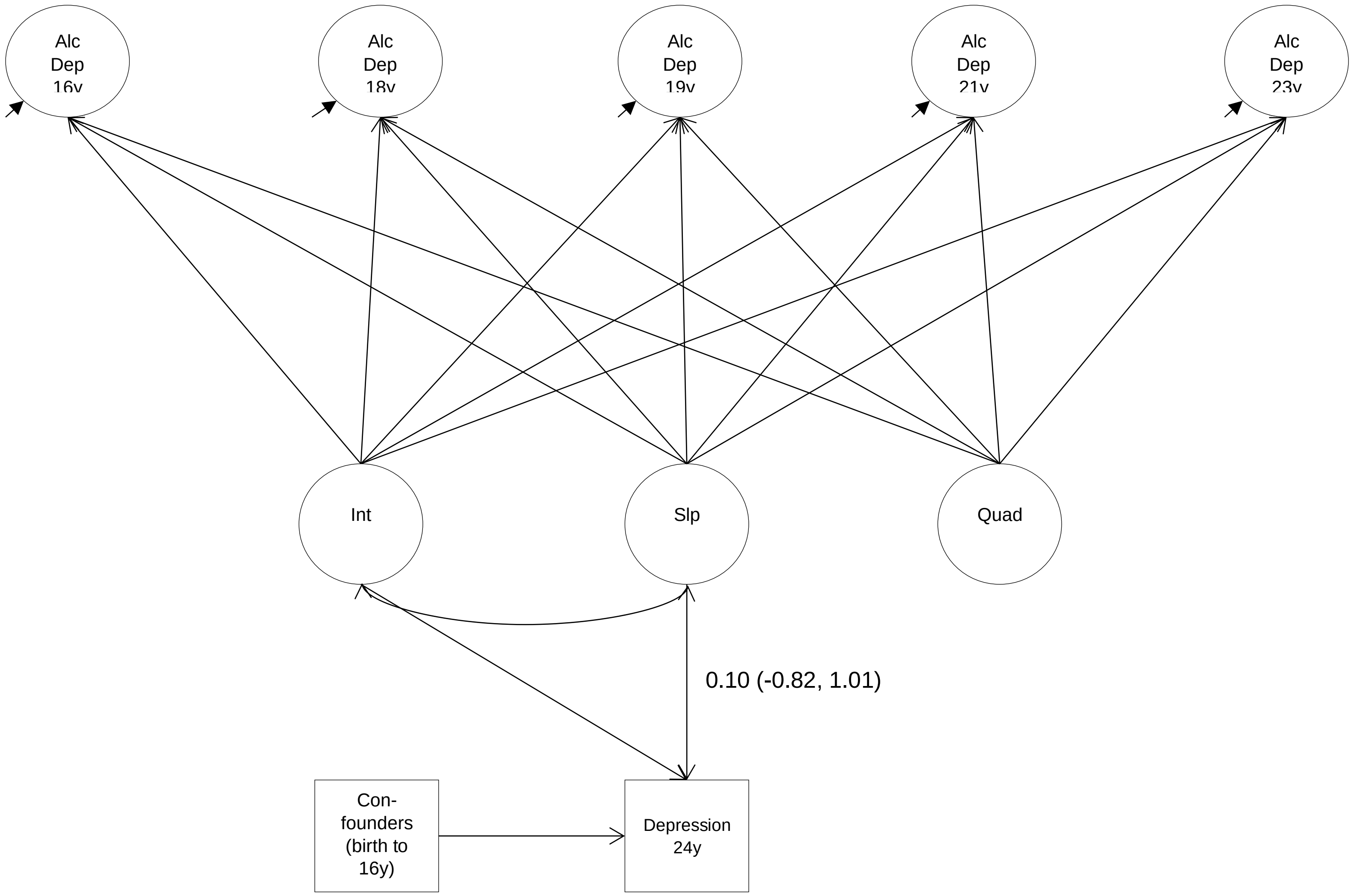

Sensitivity analyses

Sensitivity analyses included: i) regressing depression on the latent intercept fixed at each age of the growth curve for consumption and dependence; ii) additionally adjusting for household income, crowding, social class and ethnicity; and iii) rerunning analyses using those with complete data on the outcome.

To aid interpretation of the lack of association between the linear slope for alcohol consumption and dependence with depression (after adjusting for confounders), we regressed depression on levels of alcohol consumption/ dependence at each age of the growth curve (age 17 to 22 years) to examine whether the strength of association changed across late adolescence and early adulthood. Supplementary Table 11 shows that although the association for alcohol consumption weakens slightly over time and changes from positive to negative (from age 20 years), there was no evidence for an association between alcohol consumption at any age (latent intercept fixed at age 17 to 22 years) with depression at age 24. Supplementary Table 12 shows that the association between alcohol dependence and depression was also consistent across this developmental period. These analyses (associations with moving latent intercept) and the lack of association with the linear slope (shown in main text) both show that the associations between alcohol consumption/ dependence and depression remain constant over time.

Supplementary Table 11. Association between levels of alcohol consumption at each age of the growth curve (latent intercept fixed at age 17 to 22 years) and depression at age 24 years using weighted data; *N* = 3902

Levels of alcohol consumption (latent intercept)	Probit coefficient (95% CI)	p value
Age 17	0.01 (-0.03, 0.05)	0.66
Age 18	0.01 (-0.04, 0.05)	0.80
Age 19	0.001 (-0.04, 0.05)	0.97
Age 20	-0.004 (-0.05, 0.04)	0.85
Age 21	-0.01 (-0.06, 0.04)	0.67
Age 22	-0.02 (-0.06, 0.03)	0.50

Supplementary Table 12. Association between levels of alcohol dependence at each age of the growth curve (latent intercept fixed at age 17 to 22 years) and depression at age 24 years using weighted data; *N* = 3902

Levels of alcohol dependence (latent intercept)	Probit coefficient (95% CI)	p value
Age 17	0.21 (0.11, 0.31)	<0.001
Age 18	0.21 (0.11, 0.32)	<0.001
Age 19	0.21 (0.11, 0.32)	<0.001
Age 20	0.21 (0.10, 0.31)	<0.001
Age 21	0.20 (0.10, 0.31)	<0.001
Age 22	0.20 (0.09, 0.30)	<0.001

Supplementary Table 13 shows results from further sensitivity analyses for associations between growth factors for alcohol consumption and dependence with depression at age 24 years. First, we adjusted for additional demographic confounders (household income, crowding, social class and ethnicity; described in Supplement under 'Detail on the pattern and correlates of missing data) which may confound associations between alcohol and depression, and are also strong predictors of being missing from the analysis sample. These analyses therefore strengthen our assumption of adjusting for all known confounders, and also our assumption that data are missing at random conditional on exogenous variables in the analysis model. Conclusions from these analyses were unchanged.

Second, we re-ran associations using those with complete data on the outcome (depression at age 24) in addition to confounders, which resulted in a drop in the sample size from 3902 to 2222. Despite this drop in sample size, results were very similar to those presented in the main text, and conclusions remained unchanged.

Supplementary Table 13. Sensitivity analyses for associations between growth factors for alcohol consumption and dependence with depression at age 24 years; showing unstandardised probit coefficient (95% confidence interval)

	Additionally adjusting for demographic confounders[2]; *N* = 3545	Using those with complete outcome data[3]; *N* = 2222
Alcohol consumption		
Latent intercept (18 years)	-0.01 (-0.06, 0,03); p=0.58	-0.01 (-0.06, 0.03); p= 0.57
Linear slope[1]	-0.07 (-0.49, 0.36); p=0.76	-0.11 (-0.51, 0.30); p=0.60
Alcohol dependence		
Latent intercept (18 years)	0.12 (0.004, 0.23); p=0.042	0.13 (0.019, 0.24); p=0.021
Linear slope[1]	0.44 (-0.49, 1.38); p=0.35	0.11 (-0.83, 1.04); p=0.83

[1]All analyses for the linear slope adjust for the latent intercept (at age 18 years); [2]confounders included sex, housing tenure, maternal education, maternal depressive symptoms, parents' alcohol use, conduct problems, being bullied, cannabis and cigarette use, depressive symptoms at age 16 years, household income, crowding, social class and ethnicity; [3]restrciting sample to those with complete data on the outcome (depression at age 24) and adjusting for confounders used in the main text (sex, housing tenure, maternal education, maternal depressive symptoms, parents' alcohol use, conduct problems, being bullied, cannabis and cigarette use, depressive symptoms at age 16 years)

References

1 Fluharty M, Taylor AE, Grabski M, Munafò MR. The Association of Cigarette Smoking With Depression and Anxiety: A Systematic Review. *Nicotine Tob Res* 2017; **19**: 3–13.

2 Lev-Ran S, Roerecke M, Le Foll B, George TP, McKenzie K, Rehm J. The association between cannabis use and depression: a systematic review and meta-analysis of longitudinal studies. *Psychol Med* 2014; **44**: 797–810.

3 Shore L, Toumbourou JW, Lewis AJ, Kremer P. Review: Longitudinal trajectories of child and adolescent depressive symptoms and their predictors - a systematic review and meta-analysis. *Child Adolesc Ment Health* 2018; **23**: 107–20.

4 Hammerton G, Murray J, Maughan B, *et al.* Childhood Behavioural Problems and Adverse Outcomes in Early Adulthood: a Comparison of Brazilian and British Birth Cohorts. *J Dev Life Course Criminol* 2019; **5**: 517–35.

5 Goodman SH, Rouse MH, Connell AM, Broth MR, Hall CM, Heyward D. Maternal depression and child psychopathology: a meta-analytic review. *Clin Child Fam Psychol Rev* 2011; **14**: 1–27.

6 Mathers M, Toumbourou JW, Catalano RF, Williams J, Patton GC. Consequences of youth tobacco use: a review of prospective behavioural studies. *Addiction (Abingdon, England)* 2006; **101**: 948–58.

7 Moore SE, Norman RE, Suetani S, Thomas HJ, Sly PD, Scott JG. Consequences of bullying victimization in childhood and adolescence: A systematic review and meta-analysis. *World J Psychiatry* 2017; **7**: 60.

8 Melotti R, Heron J, Hickman M, Macleod J, Araya R, Lewis G. Adolescent alcohol and tobacco use and early socioeconomic position: the ALSPAC birth cohort. *Pediatrics* 2011; **127**. DOI:10.1542/PEDS.2009-3450.

9 Heron J, Maughan B, Dick DM, *et al.* Conduct problem trajectories and alcohol use and misuse in mid to late adolescence. *Drug Alcohol Depend* 2013; **133**: 100.

10 Hammerton G, Edwards AC, Mahedy L, *et al.* Externalising pathways to alcohol-related problems in emerging adulthood. *Journal of Child Psychology and Psychiatry* 2020; **61**: 721–31.

11 Joinson C, Kounali D, Lewis G. Family socioeconomic position in early life and onset of depressive symptoms and depression: a prospective cohort study. *Soc Psychiatry Psychiatr Epidemiol* 2017; **52**: 95–103.

12 Cox JL, Holden JM, Sagovsky R. Detection of postnatal depression. Development of the 10-item Edinburgh Postnatal Depression Scale. *Br J Psychiatry* 1987; **150**: 782–6.

13 Goodman A, Goodman R. Strengths and difficulties questionnaire as a dimensional measure of child mental health. *J Am Acad Child Adolesc Psychiatry* 2009; **48**: 400–3.

14 Seaman SR, White IR, Copas AJ, Li L. Combining Multiple Imputation and Inverse-Probability Weighting. *Biometrics* 2012; **68**: 129–37.

15 Khadjesari Z, White IR, McCambridge J, *et al.* Validation of the AUDIT-C in adults seeking help with their drinking online. *Addiction Science and Clinical Practice* 2017; **12**: 1–11.

16 Hammerton G, Mahedy L, Murray J, *et al.* Effects of Excessive Alcohol Use on Antisocial Behavior Across Adolescence and Early Adulthood. *J Am Acad Child Adolesc Psychiatry* 2017; **56**: 857–65.
